# Variation of the element composition of municipal sewage sludges in the context of new regulations on phosphorus recovery in Germany

**DOI:** 10.1186/s12302-022-00658-4

**Published:** 2022-09-05

**Authors:** Theresa Constanze Sichler, David Montag, Matthias Barjenbruch, Tatjana Mauch, Thomas Sommerfeld, Jan-Hendrik Ehm, Christian Adam

**Affiliations:** 1 BAM Bundesanstalt Für Materialforschung Und -Prüfung, Unter den Eichen 87, 12205 Berlin, Germany; 2 ISA Institute for Environmental Engineering, RWTH Aachen University, Mies-van-der-Rohe-Str. 1, 52074 Berlin, Germany; 3 Technical University Berlin, Gustav-Meyer-Allee 25, 13355 Berlin, Germany

**Keywords:** Wastewater, Sewage sludge, Phosphorus elimination, Phosphorus recovery, Elemental variations

## Abstract

Phosphorus (P) recovery is obligatory for all sewage sludges with more than 20 g P/kg dry matter (DM) from 2029 in Germany. Nine wastewater treatment plants (WWTPs) were chosen to investigate variations of phosphorus contents and other parameters in sewage sludge over the year. Monthly sewage sludge samples from each WWTP were analyzed for phosphorus and other matrix elements (C, N, H, Ca, Fe, Al, etc.), for several trace elements (As, Cr, Mo, Ni, Pb, Sn) and loss of ignition. Among the nine WWTPs, there are four which have phosphorus contents both above and below the recovery limit of 20 g/kg DM along the year. Considering the average phosphorus content over the year, only one of them is below the limit. Compared to other matrix elements and parameters, phosphorus fluctuations are low with an average of 7% over all nine WWTPs. In total, only hydrogen and carbon are more constant in the sludge. In several WWTPs with chemical phosphorus elimination, phosphorus fluctuations showed similar courses like iron and/or aluminum. WWTPs with chamber filter presses rather showed dilution effects of calcium dosage. As result of this study, monthly phosphorus measurement is highly recommended to determine whether a WWTP is below the 20 g/kg DM limit.

**Graphical Abstract:**

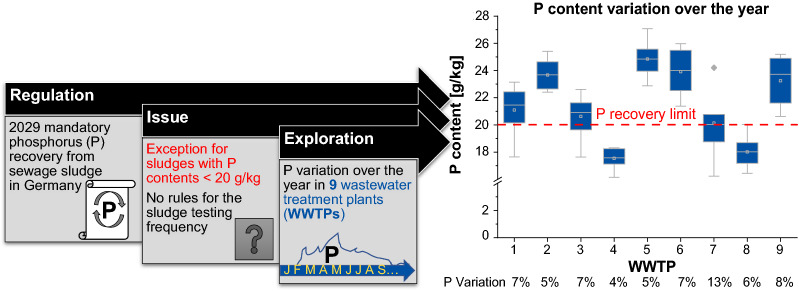

**Supplementary Information:**

The online version contains supplementary material available at 10.1186/s12302-022-00658-4.

## Background

Municipal wastewater treatment plants (WWTPs) have the original purpose to reduce epidemics and water-borne diseases by a reduction of pathogens and an interruption of the fecal–oral route. New challenges concern the recovery of nutrients, especially the element phosphorus, whose role is gaining importance in the European Union. Municipal wastewater is a secondary source for phosphorus and was already named “the untapped resource” in the UN World Water Development Report in 2017 [34]. Against the background of limited resources and the highly anthropogenic influenced nutrient cycles more circularity in food systems and phosphorus recovery are demanded by Adhya et al. [6] in their report “our phosphorus future” with contributions from 40 international scientists. Moreover, effective phosphorus removal from wastewater is essential to protect water resources from the impacts of eutrophication [35].

There are two main options for phosphorus elimination during wastewater treatment. WWTPs with chemical focus use different precipitation agents like iron or aluminum compounds [8]. WWTPs with enhanced biological phosphorus removal (EBPR) stimulate phosphate accumulating organisms which accumulate polyphosphates as energy source [20]. However, to ensure low effluent phosphorus levels and to balance inhibitory factors such as chemicals, certain toxic metals, and other factors [41], many EBPR plants use chemical precipitation in addition. Under anaerobic conditions, polyphosphates undergo a release, hence a notable amount of phosphorus is separated from the sludge and sent back to the influent. Especially struvite precipitation processes use this anaerobic release for phosphorus recovery. Although polyphosphates can account for up to 80% of the total phosphorus load in EBPR plants [39], they are not completely released which is reflected in the low possible struvite recovery rates mostly < 30% if applied before sludge thickening [12].

During the phosphorus elimination, WWTPs transfer dissolved phosphorus from wastewater to sewage sludge. Before disposal, sewage sludge is stabilized to improve dewatering and reduce the volume and the odor nuisance [13]. Anaerobic digestion is widespread in medium and large German WWTPs and provides digester gas as energy resource [9]. Aerobic stabilization usually needs larger surfaces to provide sufficient aeration and is therefore more common in smaller WWTPs. Agricultural sewage sludge utilization (disposal of stabilized sewage sludge in the agriculture for fertilization or soil improving reasons) becomes continuously less important as disposal path in Germany and other Central European countries. In the Netherlands and Switzerland, sewage sludge is completely incinerated [19]. In Germany, 74% of the sewage sludge is incinerated, 17% is utilized in agriculture and 9% disposal is unknown or covered by landscaping and other options [15]. However, sewage sludge ash is not suitable as fertilizer due to the low phosphorus plant availability [21].

Due to the high dependance on phosphorus imports, there is political will to push phosphorus recovery in Europe. In 2014, the EU added phosphate rock to the list of critical raw materials for the first time [17]. The European fertilizers ordinance was amended in 2019 to support circular economy by accepting several phosphorus recycling products [3]. This harmonization is intended to provide better market access for secondary fertilizers and more clarity about the products. The European sewage sludge directive [1] is currently in evaluation especially regarding the future of agricultural sewage sludge utilization as a priority area under the Circular Economy Action Plan [11].

In 2017, the German sewage sludge ordinance was amended to reduce agricultural sewage sludge utilization and to enhance phosphorus recovery [4]. From 2029, all WWTPs must recover phosphorus if their sewage sludge contains more than 20 g/kg DM. The total phosphorus content is analyzed in sewage sludge in the legal sense. By definition of German legislation this is the sludge after completed wastewater treatment directly before disposal (mostly stabilized sludge after dewatering). As precipitation technologies with struvite or brushite recovery mostly recover from sludge water or digested sludge before dewatering, these methods are not covered by the German legislation. Phosphorus recovery by German legal definition is recovery from dewatered sewage sludge (minimum 50% recovery prescribed or remaining phosphorus content in sludge after recovery < 20 g/kg DM) or from sewage sludge ash (minimum 80% recovery prescribed). Instead of an enhanced technical phosphorus recovery, agricultural sewage sludge utilization will still be possible for WWTPs up to 50,000 population equivalents (P.E.).

Switzerland was the first European country with a mandatory phosphorus recovery [37] from 2026. There is no exception for sewage sludges that fall below a certain phosphorus content but for small WWTPs with less than 1000 P.E. [7].

Austria aims to recover phosphorus at a level of 65–80% of the produced sewage sludge by 2030. The focus for phosphorus recovery shall be on WWTPs with more than 0.8 g phosphorus per P.E. and year. However, these aims are only goals from the 2017 Federal Waste Management Plan without legal binding. [10].

At the moment, both the German sewage sludge ordinance and the related enforcement guide contain no regulations for the frequency or statistic evaluation methods of phosphorus measurements to enable compliance with the German ordinance [4]. It is therefore for several WWTPs unclear whether they are affected by the recovery obligation or not. An evaluation of sewage sludge data from 1478 German WWTPs showed that 40% of the WWTPs have sewage sludges with phosphorus contents below 20 g/kg DM which is equal to 30% of the sludge [30]. However, phosphorus values per plant were single annual values to maximum four values per year. This study aims to investigate the variation of phosphorus and other parameters in municipal sewage sludge to define more precisely when a plant will safely fall below a certain level of phosphorus. For this reason, nine WWTPs with phosphorus contents at about 20 g/kg DM were chosen for a monthly sewage sludge sampling.

## Material and methods

Nine WWTPs were chosen for the measurement campaign. If possible, the operators took monthly samples from dewatered sewage sludge (sludge after aerobic or anaerobic stabilization and dewatering) and sent them to BAM for sample preparation and analysis. For a legally conforming phosphorus analysis according to AbfKlärV [4], both sampling and analysis must be performed by a notified laboratory. These laboratories are accredited for the performed methods and must successfully participate in annual interlaboratory comparisons. Although it was not possible to perform representative sampling in the present study, for each monthly sample several samples (different centrifuge lines or chamber filter presses) have been mixed up and rejuvenated at the BAM. Sampling did not start for all WWTPs at the same time, but all samples were taken in the period July 2020 to October 2021.

### WWTP selection

The WWTPs were specifically chosen for their phosphorus contents in the sewage sludge at about 20 g/kg DM (German recovery limit). They are different from many aspects such as capacity, wastewater treatment and sewage sludge treatment (Table 1).

**Table 1:** Wastewater treatment plants for measurement campaign

No.	Capacity [P.E.]	Treated waste-water^A^ [m^3^]	Sludge stabilization	P elimination	Sludge dewatering
a	> 1,000,000	84,100,000 ± 5%	None	EBPR, FeCl_3_	Centrifuge
b	80,000	2,300,000 ± 2%	Anaerobic	FeCl_3_, AlCl_3_	Chamber filter press
c	200,000	7,100,000 ± 11%	Anaerobic	FeCl_3_, AlCl_3_	Chamber filter press
d	75,000	2,200,000 ± 11%	Anaerobic	EBPR, FeCl_3_	Chamber filter press
e	40,000	2,400,000 ± 21%	Simultaneously aerobic	FeCl_3_, Na aluminate	Centrifuge
f	14,700	1,300,000 ± 28%	Simultaneously aerobic	Na aluminate	Centrifuge
g	17,500	1,200,000 ± 23%	Simultaneously aerobic	EBPR, FeCl_3_	Centrifuge
h	68,000	3,500,000 ± 23%	Anaerobic	EBPR, FeCl_3_	Chamber filter press
i	98,000	12,800,000 ± 17%	Anaerobic	FeCl_3_	Chamber filter press

*P.E.* population equivalents

^a^Data from 2008–2018 by the environmental portal Thru.de [33]

Data are reported every 2 years

Standard deviation of the two-yearly reported volumes is shown as ± range

The WWTPs have capacities of 14,700 P.E. to more than one million P.E. To identify them, WWTPs were numbered by letters a to i. Five (b, c, d, h, i) of the nine WWTPs perform anaerobic sewage sludge stabilization combined with a chamber filter press for dewatering. Three WWTPs (e, f, g) perform simultaneously aerobic stabilization followed by dewatering by centrifuges. One WWTP (a) has no stabilization step, and the sewage sludge is directly burned after dewatering. The WWTPs treat about 1 to more than 80 million m^3^ yearly. Especially the smaller WWTPs show higher variability of the wastewater volume with standard deviations of more than 20%.

There are four EBPR WWTPs which have either anaerobic (WWTP d, h), aerobic (WWTP g) or no sewage sludge stabilization (WWTP a). The other five WWTPs are equipped with chemical phosphorus elimination. FeCl_3_ is most common and used in all WWTPs except for WWTP f, which uses sodium aluminate as precipitation agent.

### Sample preparation and analysis

All (*n* = 101) sewage sludge samples were freeze-dried (STERIS plc, Derby, United Kingdom) and ground in a disc mill (Eaton Industries, Bonn, Germany) for further analysis. The sample size was about 500–1000 g fresh matter (30% to 40% dry matter). Thermal drying was avoided to reduce nitrogen losses during heating. Residual moisture was determined with triplicates by a moisture balance (Kilomatic, Hannover, Germany) to receive DM results.

Organic content was analyzed by the loss of ignition at 550 °C. The samples were annealed until mass constancy was reached (1.5 h for sludges with C < 250 g/kg, 3 h for samples with C > 300 g/kg). Loss of ignition was determined in a muffle furnace (Nabertherm, Lilienthal, Germany) as triplicates in corundum crucibles with a respective weight of 5000 mg.

Carbon, nitrogen, and hydrogen were analyzed by elemental analysis (Elementar Analysensysteme, Langenselbold, Germany). For this method, samples were pressed as pellets covered by tin foil. At least three independent pellets per sample (triplicates) were pressed with a respective weight of about 80 mg. The process principle is based on catalytic combustion of the pellets under temporary oxygen supply and high temperature (960 °C), subsequent post-combustion at 900 °C and chemical reduction of NO_x_ to N_2_ at 830 °C. The combustion gases CO_2_, H_2_O and N_2_ are purified and separated from each other by specific adsorption columns for CO_2_ and H_2_O. N_2_ is measured without prior absorption. By heating these adsorption columns sequentially, the gases were analyzed by thermal conductivity. The factory calibration for the elements C, H and N is checked by ethylenediaminetetraacetic acid (EDTA) as reference on each measuring day. Blanks (empty tin foil) and references (EDTA) were measured after 20 pellets.

For other matrix (P, Ca, Mg, K, S, Fe, Al, Na, Cu, Zn, Mn) and trace elemental analysis (As, Cr, Mo, Ni, Pb, Sn), samples were digested with aqua regia in a microwave (MLS, Leutkirch, Germany) according to DIN EN ISO 54321:2021.^1^ At least two digestions per sample (duplicates) were performed with a respective weight of 500 mg. In case of higher differences of the measured phosphorus values (ICP-OES RSD > 5%), further replicates were digested. Digestion solutions were filtered (LABSOLUTE type 1005, 12–15 µm) and filled up to 50 ml with distilled water.

Phosphorus solubility was tested by extraction in neutral ammonium citrate (NAC) according to the EU fertilizers regulation from 2002 [2]. For each WWTP, one to three sewage sludge samples were extracted in NAC as duplicates. Extracts were filtrated (LABSOLUTE type 2015, 5–8 µm), made up to 50 ml and measured with ICP-OES. Measurement was done with ICP-OES (Thermo Fisher, Waltham, USA) based on a five-point daily calibration with matrix adjustment for most elemental analysis and with P_NAC_ standards for P_NAC_ values. Each digestion solution was analyzed in three runs. ICP-OES measurement was done based on DIN EN ISO 11885:2009. Certified reference sewage materials (sewage sludges: product IDs CRM029-50G and CRM031-40, Sigma-Aldrich, Laramie, USA) were analyzed each day of measurement. An overview of the certified values and the range of recovery rates plus determination limits by calibration can be found in Additional file 1: Table S1.

## Results

The analyzed sewage sludges originate from different WWTPs with differing sludge and wastewater treatment. As the moisture content in the dried samples was determined after the sample digestion, all results in this study are related to the DM.

### Average sewage sludge composition

**Fig. 1:**
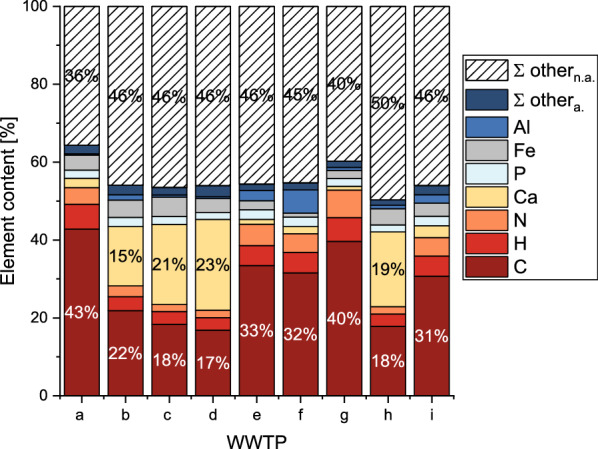
Average sludge composition (out of all monthly samples) of 9 wastewater treatment plants (WWTPs)

Regarding the sample matrix, carbon and calcium contents are most different in the sewage sludges (Fig. 1). All sludge samples can be characterized by high carbon contents (316–428 g/kg) or high calcium contents (152–236 g/kg). The carbon content in the sludges of WWTP b, c, d, and h is about half of the other sludges with 170–220 g/kg. At the same time, the calcium content in the carbon-rich sludges is only 10–30 g/kg. According to the criteria for the selection of the WWTPs, average phosphorus contents are comparably similar among all nine sewage sludges (17.5–24.8 g/kg).

Σ other_a._: share of other analyzed elements (Mg, Ka, S, Na, Cu, Zn, Mn, As, Cr, Mo, Ni, Pb, Sn).

Σ other_n.a._: share of not analyzed elements (O, Si, etc.)

Hydrogen contents reach 30–60 g/kg, nitrogen 20–70 g/kg and iron 10 to 50 g/kg. Most sludges have low aluminum levels < 15 g/kg but sludge e, f and i show higher contents up to 60 g/kg for WWTP f (Table 2). The total share of analyzed elements (Σ C, N, H, Ca, Mg, Ka, P, S, Fe, Al, Na, Cu, Zn, Mn, As, Cr, Mo, Ni, Pb, Sn) was different reaching from 50% for WWTP i up to 64% for WWTP a. Lower proportional matrix elements were sodium (Na), zinc (Zn), manganese (Mn), and copper (Cu) at about 200 mg/kg to max. 1300 mg/kg.

**Table 2:** Average matrix element contents in g/kg (of all samples) in sludges of 9 wastewater treatment plants (WWTPs)

WWTP	a	b	c	d	e	f	g	h	i
n	11	10	13	10	12	12	9	12	12
C^1^	428.0	218.4	183.4	170.1	338.1	315.5	397.4	178.5	324.4
Ca	24.0	152.1	205.4	235.5	12.4	18.9	9.9	191.7	29.9
H^1^	64.6	37.0	33.5	33.2	52.7	53.4	61.7	31.8	50.9
N^1^	42.5	27.7	18.4	19.4	55.4	47.9	70.8	19.1	50.0
P	21.1	23.7	20.6	17.5	24.8	23.9	20.1	18.0	23.3
Fe	38.4	44.0	49.8	36.4	23.6	10.6	20.6	41.3	32.8
Al	3.5	14.1	5.5	4.5	26.9	59.2	7.4	9.0	21.1
S	16.1	9.5	7.1	18.7	5.5	5.0	6.2	4.0	9.7
Mg	2.5	9.0	8.5	6.8	3.7	5.1	3.5	6.0	6.2
K^1^	1.6	3.3	1.9	1.6	4.7	5.1	4.8	2.2	3.9
Na	0.7	0.9	0.6	0.9	1.0	1.3	0.6	0.6	0.6
Zn	0.6	0.8	0.5	0.4	1.0	1.1	0.7	0.6	0.8
Mn	0.3	0.3	0.3	0.2	0.3	0.3	0.2	0.3	0.8
Cu^1^	0.4	0.2	0.2	0.2	0.2	0.2	0.2	0.2	0.3
o.c	80%	40%	30%	26%	65%	62%	73%	26%	60%
$\frac{\mathrm{C}}{\mathrm{o}.\mathrm{c}.}$	54%	55%	60%	65%	52%	51%	55%	70%	54%
$\frac{{\mathrm{P}}_{\mathrm{NAC}}}{\mathrm{P}}$ ^2^	80%	91%	93%	95%	90%	76%	70%	91%	84%
$\frac{Fe+Al}{P}$	1.2	1.7	1.6	1.4	1.8	3.1	1.0	1.8	1.8

*n* number of samples; *o.c.* organic content as loss of ignition (550 °C)

^1^Number of analyzed samples for C, H, N, K and Cu is lower than n in some WWTPs (*n* = 7–11). For detailed information check the Additional file which contains measurement data for each sample per WWTP a-i (Additional file 1: Fig. S1–S9)

^2^Share of phosphorus load which is NAC-extractable (average of one to three samples per WWTP) and molar ration of phosphorus and precipitation agents iron and aluminum (average of all samples), also check Additional file 1: Fig. S10 for more information

The organic content (as loss of ignition at 550 °C) in the sludge samples was about twice the carbon content for WWTP a, b, e, f, g, and i. However, carbon contents were higher in WWTP c, d, and h reaching from 60% (WWTP d) to 70% (WWTP h) of the loss of ignition. These sewage sludges show high calcium contents, too. All element results (average contents and RSD) by WWTP and sample number can be found in Additional file 1: Tables S2–S19.

As P_NAC_ was analyzed only for single samples, it was not possible to build annual averages for the plant available phosphorus. All sludges showed P_NAC_ values > 65% of the total P content. WWTP b, c, d, e, and h had particularly high shares of 87% to 100% P_NAC_. The lowest P_NAC_ at 66% was found in a sludge sample from WWTP g. The average molar ratio of the precipitation agents iron and aluminum $\frac{Fe+Al}{P}$_Average_ (total iron and aluminum contents) to phosphorus was between 1.0 (WWTP g) and 3.1 (WWTP f). Most sewage sludges showed an average molar ratio of 1.6–1.8. The molar ratios in the samples that were analyzed for P_NAC_ were mostly like the average ratio (less than 15% deviation for all single samples). Detailed data on molar ratios and P_NAC_ can be found in Additional file 1: Table S20.

Due to some measurement problems for single days at the ICP-OES (potassium peaks not detected) and elemental analysis (unsatisfactory day factor for hydrogen), for some elements there are less values than sewage sludge samples. However, except from hydrogen for WWTP c (only 5/13 samples analyzed), this applies for only few matrix elements (C, H, N, K, Cu) in single samples of some WWTPs. For the trace elements As and Sn, the calibration standards showed declining intensity by 50% after several weeks. Therefore, there are less values than samples for these elements for all WWTPs; for WWTPs f, g, and i no valid values can be reported. Arsenic and molybdenum (Mo) were the lowest analyzed trace elements (Table 3) with contents of less than 1 mg/kg (As at WWTP d) to 16 mg/kg (Mo at WWTP i).

**Table 3:** Average trace element contents in mg/kg (of all samples) in sludges of 9 wastewater treatment plants (WWTPs)

WWTP	a	b	c	D	e	f	g	h	i
n	11	10	13	10	12	12	9	12	12
As^1^	5	3	1	< 1	4			2	
Mo	5	4	2	3	5	5	3	1	16
Sn^1^	13	16	14	8	23			13	
Ni	15	27	15	13	20	30	20	19	40
Cr	20	28	22	11	36	38	27	29	50
Pb	22	26	24	11	51	52	42	24	41

*n* number of samples

^1^No arsenic and tin values for WWTP f, g and i. Number of analyzed samples is lower for several WWTPs. For detailed information check the Additional file which contains measurement data for each sample per WWTP a-i (Additional file 1: Fig. S1–S9)

Tin was at average 8 mg/kg (WWTP d) to 23 mg/kg (WWTP e), nickel (Ni) at 13 mg/kg (WWTP d) to maximum 40 mg/kg (WWTP i). Chromium (Cr) was reaching from 11 mg/kg (WWTP d) to 50 mg/kg (WWTP i) and lead (Pb) from 11 mg/kg (WWTP d) to 52 mg/kg (WWTP e). Higher contents of heavy metals occurred especially in WWTP e, f, and i whereas WWTP d showed the lowest contents for most measured trace elements (except from molybdenum).

### Variation of P-content in sewage sludges during the year

The sewage sludges from WWTP a, c, g and h were above and below the “recovery limit”, with plant h only once exceeding the limit of 20 g/kg. In contrast, the sewage sludges from WWTP b, e, f and i were steadily above the limit with average values between 23 and 25 g/kg. All monthly phosphorus values by WWTP are shown in Additional file 1: Fig. S1.

**Fig. 2:**
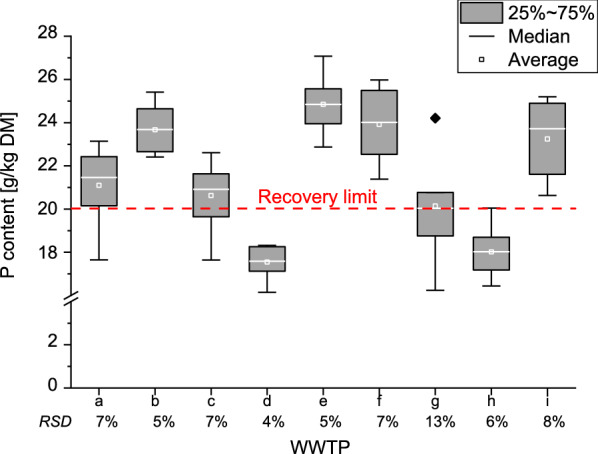
Phosphorus content ranges for sludges from 9 wastewater treatment plants (WWTPs). Relative standard variations below

For a brief statistical overview of the measured values, the results are also presented as boxplots (Fig. 2). In most cases, median and average phosphorus contents were almost congruent. WWTP a, c and i showed higher deviations. The median P content for the sludge from all these WWTPs was higher than the average.

A special case is WWTP g which shows particularly higher P contents (+ 28% compared to the mean of the other values) in the first and in the last sewage sludge sample. The black mark above the box of WWTP g in Fig. 2 does not represent outliers, but two single values towards the beginning and end of the measurement series lying on top of each other. There is also the fact that WWTP g has a gap between the first and second sample of nearly four months due to a defect of the centrifuges.

WWTP d has the most constant phosphorus content with an RSD (relative standard deviation) for phosphorus over the whole year of only 4%. WWTP b and e also show low variability of about 5% RSD. On the contrary, WWTP a, g, and i show the highest variation in the phosphorus contents with 7% to 13% (WWTP g) RSD.

## Discussion

Due to the pandemic COVID-19 which reached its first climax in Germany during spring 2020, for several WWTPs sampling was not possible between March and May 2020. Generally, 2020 was not a standard year due to the pandemic especially regarding movement patterns of the population. However, none of the regarded WWTPs were in special tourist areas. Moreover, the size of at least 14,700 P.E. means a certain stability in the wastewater treatment and the WWTP drainage areas. Four of the nine WWTPs were chosen from the sewage sludge reports^2^ from 2016 and 2017. A comparison of the phosphorus contents from 2020 and 2016 or 2017 shows similar values. For all these reasons, the pandemic should not have significantly affected the measurement campaign and the results can be regarded as it would have been normal conditions.

### Discussion of elemental contents

The sewage sludge samples were extensively characterized by the measurements of C, N, H, Ca, Mg, Ka, P, S, Fe, Al, Na, Cu, Zn, Mn, As, Cr, Mo, Ni, Pb, Sn. The capture rate of 50–64% of the total sample matrix is mostly reached by the elements C, N, Ca, P, Fe, and Al which accounted for already 47–56%. This share of analyzed elements may seem low at first glance. However, other sewage sludges show similar values with 40–45% analyzed matrix according to Suanon et al. [32] and with 51% analyzed matrix (covered by C, N, P, Fe, and Al) according to Sailer et al. [28].

It is likely that the remaining matrix is mostly oxygen, which is oxidic, carbonate or bound in the organics. Silicon was not analyzed (aqua regia digestion not suitable), but does not appear to be a major component of sewage sludge with levels < 1% [28, 36].

All nine WWTPs were chosen because of their phosphorus contents of about 20 g/kg. As phosphorus limits in the wastewater effluent are significantly stricter for WWTPs > 10,000 P.E. [5], these sludges tend to higher phosphorus loads than those of smaller WWTPs [30]. However, for the regarded WWTPs in this study, there are two main reasons for the low phosphorus contents in the sewage sludge. On the one hand, aerobically stabilized sludges show high carbon contents up to 40% which have diluting effects on the phosphorus content. On the other hand, anaerobically stabilized sewage sludges in this study are dewatered by chamber filter presses. This dewatering method is often accompanied by high calcium dosage to improve the dewaterability [26].

Today, German operators mostly use centrifuges for sewage sludge dewatering as higher dry residue is already reached by low polymer usage. Four of the five WWTPs with anaerobic sewage sludge stabilization show these high calcium contents of 150–250 g/kg compared to only 10–30 g/kg in the aerobically stabilized sludges. Low phosphorus contents are therefore a result of diluting effects and not necessarily attributed to low phosphorus loads.

This discrepancy between phosphorus content and load leads to a central point of criticism of the German sewage sludge ordinance raised several times [24]. It is questionable whether the phosphorus content is the right parameter to deal with as it is highly dependent on processes during the wastewater treatment: anaerobic stabilization, e.g., reduces organic matter thus increases the phosphorus content, calcium dosage dilutes thus reduces the phosphorus content. At the same time, phosphorus loads are constant over the time. Contrary to Germany, phosphorus influx loads instead of contents are crucial when it comes to phosphorus recovery in Switzerland [7] and Austria [10]. Some element contents like several heavy metals of the nine sewage sludges can be compared to the limits of the German [16] and European fertilizer ordinance [3] and to the average contents of agriculturally utilized sewage sludges between 2013 and 2016 [14]. The total average of all nine sewage sludges (4 of which became agriculturally utilized) is in most cases comparable to the total average contents in agricultural utilized sewage sludge from the sewage sludge reports (Table 4). Despite of nickel in one monthly sample at WWTP i, none of the regarded sludges exceeded the limits of the German or the European fertilizer ordinance throughout the campaign.

**Table 4:** Element contents values of 9 WWTPs compared to averages in ASS and to ordinance limits

		2013–2016 [14] ASS^a^	German FO limit [16]	EU FO limit (2019/1009/EU, 2019)	Average of 9 WWTPs	Maximum of 9 WWTPs	WWTP no. with maximum value
As	[mg/kg]		40	40	3	8	e
Pb	[mg/kg]	30–33	150	120	33	77	e
Cu	[mg/kg]	294–309	5000	600	219	521	a
Ni	[mg/kg]	24–26	80	50	22	64	i
Zn	[mg/kg]	773–800	4000	1500	727	1,136	e
Cr	[mg/kg]	31–34			29	56	i
N	[mg/kg]	43,800–45,200			39,020	77,680	g
P	[mg/kg]	25,600–26,600			21,460	27,080	e
K	[mg/kg]	3600–3700			3220	6240	f
Mg	[mg/kg]	5600–5800			5670	9790	b
Ca	[mg/kg]	79,800–84,300			97,760	273,890	d
o.c	[%]	52			57	80	a

*ASS* agriculturally utilized sewage sludge, *FO* fertilizer ordinance, *WWTP* wastewater treatment plant

^a^K, Mg and Ca converted from oxide values (K_2_O, MgO and CaO as alkaline content). Annual averages from the sewage sludge reports which consider all agriculturally utilized sewage sludges for the respective year

The levels of aluminum and iron in the sewage sludge samples are highly influenced by the phosphorus elimination method (Table 1). However, it must be added that AlCl_3_ is apparently sparingly used compared to FeCl_3_ in the WWTPs b and c, as they show partly lower contents than WWTPs without an Al-based phosphorus elimination (Table 2g–i).

To estimate the plant availability of phosphorus in fertilizers, there are different extraction tests established such as water solubility, citric acid solubility and solubility in neutral ammonium citrate (NAC) solution [3]. There are indications that high iron and aluminum contents in sewage sludge decrease the plant availability [29]. The relative phosphorus fertilizer availability (measured as water extractable P) is highly dependent on the molar $\frac{Fe+Al}{P}$ ratio with molar ratios between 0.7 and 1.5 according to Lemming et al. [25]. Moreover, Ca:P ratios do also affect the water solubility of phosphorus in sewage sludge according to Huang and Shenker [22].

For the regarded WWTPs P_NAC_ was not obviously only dependent on either the Fe:P or Al:P ratio nor on the phosphorus elimination. The sample with the lowest NAC solubility of 66% came from an EBPR plant (WWTP g, average P_NAC_ 70%) instead and showed the lowest molar $\frac{Fe+Al}{P}$ ratio of only 0.7. At the same time, WWTP h showed a high average P_NAC_ of 91% despite a high $\frac{Fe+Al}{P}$ of 1.8. Moreover, comparing the respective two samples per WWTP shows that higher $\frac{Fe+Al}{P}$ ratios do not necessarily mean lower P_NAC_ and vice versa (WWTP a, c, d, f, g, h, i). Generally, the $\frac{Fe+Al}{P}$ ratio is rather constant over the year and P_NAC_ seems to vary more. Unnoticed errors during sample preparation were reduced by duplicate digestions which showed low RSD_ICP-OES_ of < 1–6% (except one sample of WWTP i with RSD_ICP-OES_ = 11%).

It should therefore be discussed whether P_NAC_ reflects well the plant availability of these sewage sludges. P_NAC_ is not ideal to show the phosphorus plant availability in biosolids which is rather inversely correlated to the total content of iron and aluminum according to Elliot et al. [18]. Moreover, NAC extraction carries the risk of significantly overestimating plant availability especially for a model Fe–P based sewage sludge according to Steckenmesser et al. [31]. NAC extraction therefore seems to be no suitable method to show the plant availability of phosphorus in these sewage sludges.

### Reasons for phosphorus fluctuations in sewage sludge

**Fig. 3:**
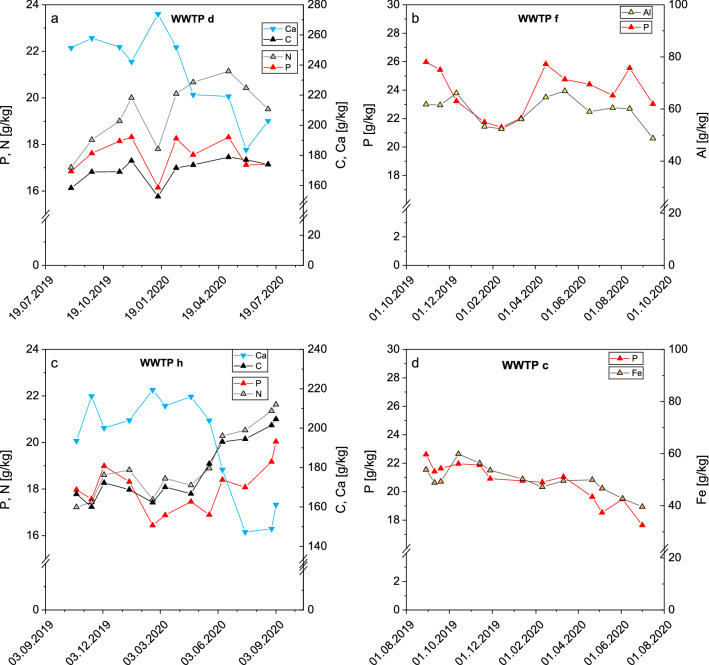
Examples for congruent trend of phosphorus and precipitation elements and opposite trend of phosphorus and calcium

The monthly sewage sludge tests at nine WWTPs have shown that phosphorus in sewage sludge is subject to natural fluctuations. One reason for these fluctuations might be higher phosphorus levels in the WWTP influent and therefore higher precipitation dosage for the phosphorus elimination. For some sewage sludges, annual patterns like that of phosphorus have been discovered for specific elements (Fig. 3). An example for such a similar annual pattern is aluminum at WWTP f and iron at WWTP c. Both WWTPs perform a purely chemical phosphorus precipitation by sodium aluminate (WWTP f) or mainly FeCl_3_ (WWTP c) which leads to consistent Fe:P or Al:P ratios.

Phosphorus fractionation in sewage sludge is dependent on the elimination focus (chemical or biological) and on the molar ratio of the precipitation elements iron and/or aluminum to phosphorus. Significant amounts of phosphorus especially in digested sewage sludge are bound in vivianite (Fe_2_(PO_4_)_3_ ∙ 8H_2_O) reaching 70–90% for high molar Fe:P ratios of 2.5 according to Wilfert et al. [38]. However, the regarded sewage sludges of this study show molar Fe:P ratios of maximum 1.4. Nevertheless, Fe–P compounds (not necessarily vivianite) are still the largest phosphorus fraction (58–99%) in digested sewage sludge according to Zhang et al. [40].

Another example is carbon and nitrogen in the sludges of the WWTPs d and h which are performing EBPR. As a result, phosphorus is largely associated with the organics. Dependent on wastewater and sludge treatment, organic phosphorus accounts for 10–30% of the total phosphorus and polyphosphates for 30–80% according to Yu et al. [39]. Apart from concentration effects, dilution effects are possible in sewage sludge especially by the high calcium addition in WWTPs b, c, d, and h. This dilution effect can be shown by the opposite course of calcium in Fig. 3.

These trends were not detected for all WWTPs. In consultation with the operators of some WWTPs it became clear that precipitating agents were partially dosed highly above the need. The reason were defects at the phosphorus measurement device. Therefore, precipitation dosage was increased to ensure low phosphorus effluent levels meeting the wastewater restrictions.

Over the year and all WWTPs, phosphorus levels were lowest in the winter between January and March and highest in the late summer between August and October (Additional file 1: Fig. S2). However, these phosphorus content differences are less than ± 10% averaged over all plants. The main precipitation agent iron (aluminum for WWTP f) shows a similar trend with higher deviation though (extreme values between 66 and 236% of the annual mean, cf. Additional file 1: Fig. S2).

Generally, phosphorus variations in the sewage sludge are low compared to many other matrix elements at most of the investigated WWTPs. Compared to the respective phosphorus variation RSD, there is no element more constant at WWTP b, for WWTP d and h only hydrogen and for WWTP e only the organic content (as loss of ignition at 550 °C).

In contrast, WWTP g with the highest phosphorus variation of 13% RSD shows many other element contents (C, H, N, Mg, Zn, Ni, organic content) with higher stability throughout the year. At the same time, WWTP g has the highest single RSD with values of more than 50% for iron and manganese. WWTP a shows less fluctuations than phosphorus for the content of carbon, nitrogen, hydrogen and the organic content and WWTP c for carbon and hydrogen (only five values for H). Regarding the treated wastewater as another measure of variability (Table 1), high influent variability (highest value at WWTP f: 28%) does not necessarily mean a higher phosphorus content variability (mean value at WWTP f: 7%).

Regarding all nine WWTPs, only hydrogen and carbon contents are more constant in the sewage sludge over the year than phosphorus (Additional file 1: Fig. S3). In total, iron and manganese show the highest variations with more than 250% of the phosphorus variation (RSD_Mn_ = 18%, RSD_Fe_ = 19%). Other matrix elements and parameters are between 100 and 200% of the phosphorus variation.

Although a comparable monitoring has not yet been performed for other countries and WWTPs, there are few investigations on seasonal changes in the composition of sewage sludge ash. Ohbuchi et al. [27] found a strong mutually complementary correlation between SiO_2_ and P_2_O_5_ and a correlation between SiO2 and CaO by monthly analysis of one Japanese sewage sludge ash. The total variation of P was very high with contents between 96 and 170 g/kg. However, they do not provide numbers for the variations as RSD although the maximum concentrations were mentioned to be irregularly high. [27]

Kasina et al. [23] found a high stability (no numbers mentioned) for the main elements phosphorus, calcium, iron, aluminum and silicon by monthly analysis of one Polish sewage sludge ash. Both studies [23, 27] report a relative decrease of phosphorus contents in summertime which was not observed in the present study (Additional file 1: Fig. S2). However, comparability with sewage sludge ash is limited since silicon exerts an increased influence here due to the greatly reduced mass.

### Recommendation for sewage sludge analysis according to the German legislation

The elemental analysis showed that phosphorus fluctuations are low compared to other elemental variations and depend on several parameters which have diluting (Ca) and concentrating (Fe, Al) effects. The WWTPs a, c, g and h showed contents above and below the 20 g/kg limit with fluctuations between 6 and 13% RSD. Several recommendations for the testing of sludges with marginal phosphorus contents can be derived from the results of the annual phosphorus variations in the investigated sewage sludges.

Sampling and phosphorus measurement should be considerably more than once a year, especially for those WWTPs that show phosphorus contents between 18 and 22 g/kg. These analyzes should happen in regular time intervals. A suitable time interval could be calculated based on the respective solids retention time of the activated sludge process and—if existing—the hydraulic retention time of the anaerobic digestion to cover different sludge flows as well as possible. If the phosphorus variations are mainly caused by diluting effects as calcium dosage, variations might still be more dynamic. However, monthly sewage sludge sampling and analysis comparable with the present study would keep the effort for WWTPs in a reasonable extent (especially smaller WWTPs still use chamber filter presses for dewatering) and would still cover the most changes in the sludge composition.

Analogous to German and European environmental legislation, the 95th percentile is common for emission control [5]. However, on the one hand in the case of phosphorus it is not a question of protection from pollutants emission but of a recovery enforcement. One the other hand, a percentile of ≥ 90% would be equal to an exceedance of zero values (*n* = 12). With a 75th percentile, three out of 12 monthly phosphorus contents could be above the limit. Regarding the present study, two WWTPs (d and h) meet this percentile. WWTP g which is above the limit by average and below by median is clearly above the limit regarding the 75th percentile.

The authors clearly recommend a percentile over a decision by average annual content because the arithmetic mean is more susceptible to single low values, hence easier to manipulate.

## Conclusions

Based on the performed monitoring of 9 WWTPs in this study, the authors recommend a monthly sewage sludge sample analysis over one year to determine whether a WWTP is affected by the German phosphorus recovery obligation or not. A safe undercut could be established if 75% of the determined phosphorus contents are below the limit of 20 g/kg DM. Thus, the WWTP would be excluded from the German phosphorus recovery duties. Regarding other European approaches for phosphorus recovery at WWTPs, the Swiss and Austrian regulations appear to be simpler and more reasonable as they refer to the WWTP capacity or the phosphorus load instead of phosphorus contents in sewage sludge. Therefore, the authors would recommend limits in recovery obligations based on nutrient loads or WWTP capacities for other countries aiming at phosphorus recovery. As critical raw material by EU listing the recovery of phosphorus from sewage sludge and/or other waste streams will become a topic possibly also regarding the currently revised European sewage sludge ordinance.

## Supplementary Information

**Additional file 1: Figure S1.** Phosphorus content variations (dry matter) in municipal sewage sludge at 9 WWTPs. **Figure S2.** Range of monthly phosphorus and iron content by annual mean during the year. **Figure S3.** Ranges of relative standard deviation (RSD) of matrix elements and organic content for the 9 WWTPs over the whole year. **Table S1.** Certified reference material sewage sludges used for analysis and calibration limits. **Table S2.** Total data for WWTP a, all analyzed values per sample. **Table S3.** Relative standard deviation data of the replicates for analyzed values of WWTP a. **Table S4.** Total data for WWTP b, all analyzed values per sample. **Table S5.** Relative standard deviation data of the replicates for analyzed values of WWTP b. **Table S6.** Total data for WWTP c, all analyzed values per sample. **Table S7.** Relative standard deviation data of the replicates for analyzed values of WWTP c. **Table S8.** Total data for WWTP d, all analyzed values per sample. **Table S9.** Relative standard deviation data of the replicates for analyzed values of WWTP d. **Table S10.** Total data for WWTP e. all analyzed values per sample. **Table S11.** Relative standard deviation data of the replicates for analyzed values of WWTP e. **Table S12.** Total data for WWTP f. all analyzed values per sample. **Table S13.** Relative standard deviation data of the replicates for analyzed values of WWTP f. **Table S14.** Total data for WWTP g. all analyzed values per sample. **Table S15.** Relative standard deviation data of the replicates for analyzed values of WWTP g. **Table S16.** Total data for WWTP h. all analyzed values per sample. **Table S17.** Relative standard deviation data of the replicates for analyzed values of WWTP h. **Table S18.** Total data for WWTP i. all analyzed values per sample. **Table S19.** Relative standard deviation data of the replicates for analyzed values of WWTP i. **Table S20.** Share of in neutral ammonia citrate (NAC) extractable phosphorus (P_NAC_) in several sewage sludge samples and molar ratios of P and potential precipitation agents Fe and Al.

## Data Availability

All evaluated data generated or analyzed during this study are included in this published article and its Additional files. Raw data can be handed out on request by the corresponding author.

## Abbreviations

DM: Dry matter
EBPR: Enhanced biological phosphorus removal
EDTA: Ethylenediaminetetraacetic acid
NAC: Neutral ammonium citrate
P.E.: Population equivalent
RSD: Relative standard deviation
WWTP: Wastewater treatment plant

## Footnotes

1 ISO 54321 was published after the sample preparations. However, there are no differences between the used German DIN EN 16174 and the ISO 54321 for microwave digestion.

2 WWTPs with agricultural sewage sludges utilization must report their nutrient and heavy metal contents annually to federal agencies in Germany which aggregate them for the public. Raw data have been requested from the agencies to identify WWTPs with marginal P contents and evaluated in detail.
