# Supplementary material for: Variation of the element composition of municipal sewage sludges in the context of new regulations on phosphorus recovery in Germany
Source: Environ Sci Eur. 2022 Sep 5;34(1):84. doi: 10.1186/s12302-022-00658-4 (PMC9442560; doi:10.1186/s12302-022-00658-4)
Supplement: Supplementary file 1 — Additional file 1: Figure S1. Phosphorus content variations (dry matter) in municipal sewage sludge at 9 WWTPs. Figure S2. Range of monthly phosphorus and iron content by annual mean during the year. Figure S3. Ranges of relative standard deviation (RSD) of matrix elements and organic content for the 9 WWTPs over the whole year. Table S1. Certified reference material sewage sludges used for analysis and calibration limits. Table S2. Total data for WWTP a, all analyzed values per sample. Table S3. Relative standard deviation data of the replicates for analyzed values of WWTP a. Table S4. Total data for WWTP b, all analyzed values per sample. Table S5. Relative standard deviation data of the replicates for analyzed values of WWTP b. Table S6. Total data for WWTP c, all analyzed values per sample. Table S7. Relative standard deviation data of the replicates for analyzed values of WWTP c. Table S8. Total data for WWTP d, all analyzed values per sample. Table S9. Relative standard deviation data of the replicates for analyzed values of WWTP d. Table S10. Total data for WWTP e. all analyzed values per sample. Table S11. Relative standard deviation data of the replicates for analyzed values of WWTP e. Table S12. Total data for WWTP f. all analyzed values per sample. Table S13. Relative standard deviation data of the replicates for analyzed values of WWTP f. Table S14. Total data for WWTP g. all analyzed values per sample. Table S15. Relative standard deviation data of the replicates for analyzed values of WWTP g. Table S16. Total data for WWTP h. all analyzed values per sample. Table S17. Relative standard deviation data of the replicates for analyzed values of WWTP h. Table S18. Total data for WWTP i. all analyzed values per sample. Table S19. Relative standard deviation data of the replicates for analyzed values of WWTP i. Table S20. Share of in neutral ammonia citrate (NAC) extractable phosphorus (PNAC) in several sewage sludge samples and molar ratios of P and potentia [file 12302_2022_658_MOESM1_ESM.pdf]

# Additional file 1

# Variation of the element composition of municipal sewage sludges in the context of new regulations on phosphorus recovery in Germany

Theresa Constanze Sichler[a,1], David Montag[b], Matthias Barjenbruch[c], Tatjana Mauch[a], Thomas Sommerfeld[a], Jan-Hendrik Ehm[b], Christian Adam[a]

[a] BAM Bundesanstalt für Materialforschung und -prüfung, Unter den Eichen 87, 12205 Berlin, Germany
[b] ISA Institute for Environmental Engineering, RWTH Aachen University, Mies-van-der-Rohe-Str. 1, 52074 Aachen
[c] Technical University Berlin, Gustav-Meyer-Allee 25, 13355 Berlin, Germany

## Contents

### Figures

Figure S1: Phosphorus content variations (dry matter) in municipal sewage sludge at 9 WWTPs ................. 3

Figure S2: Range of monthly phosphorus and iron content by annual mean during the year ........................ 4

Figure S3: Ranges of relative standard deviation (RSD) of matrix elements and organic content for the 9 WWTPs over the whole year ........................................................................................................... 4

### Tables

Table S1: Certified reference material sewage sludges used for analysis and calibration limits ..................... 5

Table S2: Total data for WWTP a, all analyzed values per sample ................................................................ 6

Table S3: Relative standard deviation data of the replicates for analyzed values of WWTP a ........................ 7

Table S4: Total data for WWTP b, all analyzed values per sample ................................................................ 8

Table S5: Relative standard deviation data of the replicates for analyzed values of WWTP b ........................ 9

Table S6: Total data for WWTP c, all analyzed values per sample ................................................................ 10

Table S7: Relative standard deviation data of the replicates for analyzed values of WWTP c ...................... 11

Table S8: Total data for WWTP d, all analyzed values per sample ................................................................ 12

Table S9: Relative standard deviation data of the replicates for analyzed values of WWTP d ...................... 13

Table S10: Total data for WWTP e. all analyzed values per sample .............................................................. 14

Table S11: Relative standard deviation data of the replicates for analyzed values of WWTP e .................... 15

Table S12: Total data for WWTP f. all analyzed values per sample ............................................................... 16

[1] Corresponding author.
*E-Mail address*: theresa.sichler@bam.de (T. Sichler).

***Additional file 1*** *- Variation of the element composition of municipal sewage sludges in the context of new regulations on phosphorus recovery in Germany*

Table S13: Relative standard deviation data of the replicates for analyzed values of WWTP f ....................17
Table S14: Total data for WWTP g. all analyzed values per sample ..............................................................18
Table S15: Relative standard deviation data of the replicates for analyzed values of WWTP g....................19
Table S16: Total data for WWTP h. all analyzed values per sample ..............................................................20
Table S17: Relative standard deviation data of the replicates for analyzed values of WWTP h....................21
Table S18: Total data for WWTP i. all analyzed values per sample ...............................................................22
Table S19: Relative standard deviation data of the replicates for analyzed values of WWTP i.....................23
Table S20: Share of in neutral ammonia citrate (NAC) extractable phosphorus ($P_{NAC}$) in several sewage sludge samples and molar ratios of P and potential precipitation agents Fe and Al ....................................24

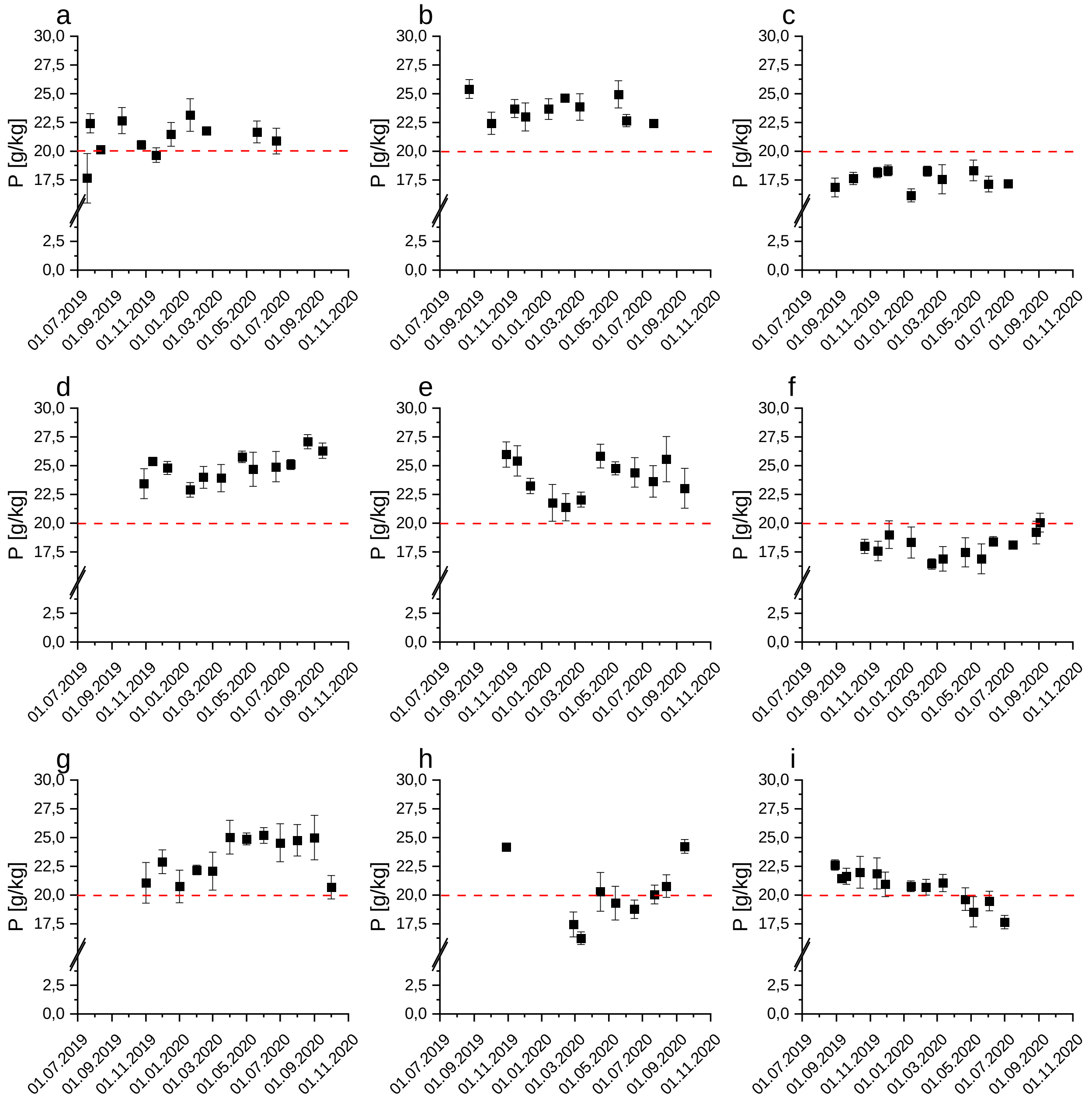


**Figure S1: Phosphorus content variations (dry matter) in municipal sewage sludge at 9 WWTPs**

The error bars in Figure S1 are shown based on the P measurement standard deviation between the duplicates (ICP-OES) and the standard deviation of the moisture analysis (triplicates). The ICP-OES coefficients of variation ($RSD_{OES}$) for all duplicates was at maximum 6%, average 2%. However, moisture contents showed higher differences with $RSD_{moist}$ up to 11%, average 3%. In general, further replicates were done in case of RSDs > 5% but due to the need of higher masses more replicates were not possible for the moisture analysis for all samples. All monthly values for the phosphorus content are also shown in Table S2, S4, S6, S8, S10, S12, S14, S16 and S18 ($6^{th}$ row).

***Additional file 1*** *- Variation of the element composition of municipal sewage sludges in the context of new regulations on phosphorus recovery in Germany*

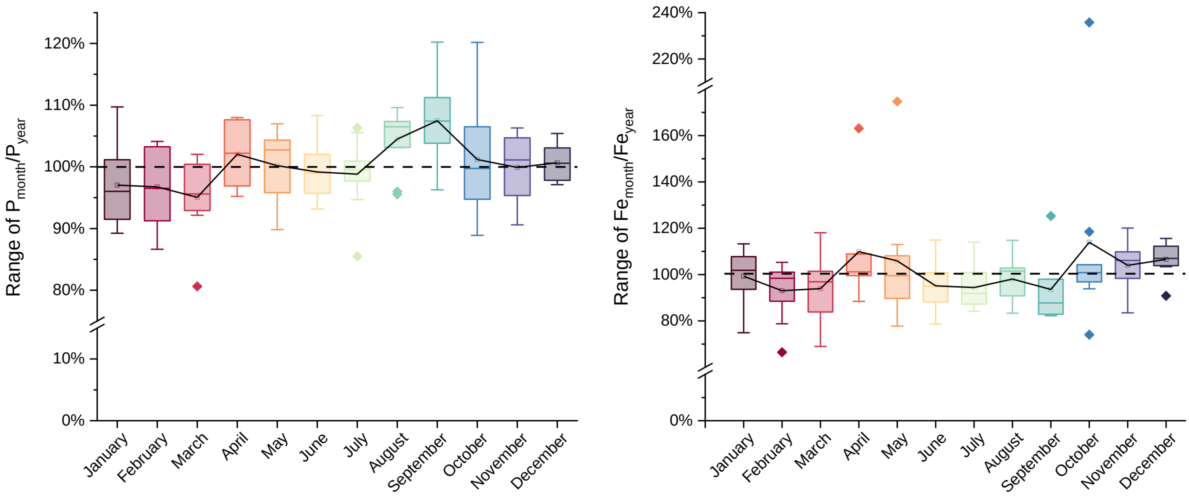


**Figure S2: Range of monthly phosphorus and iron content by annual mean during the year**

Phosphorus variations from the annual mean are also shown in table S2, S4, S6, S8, S10, S12, S14, S16 and S18 (7th row)

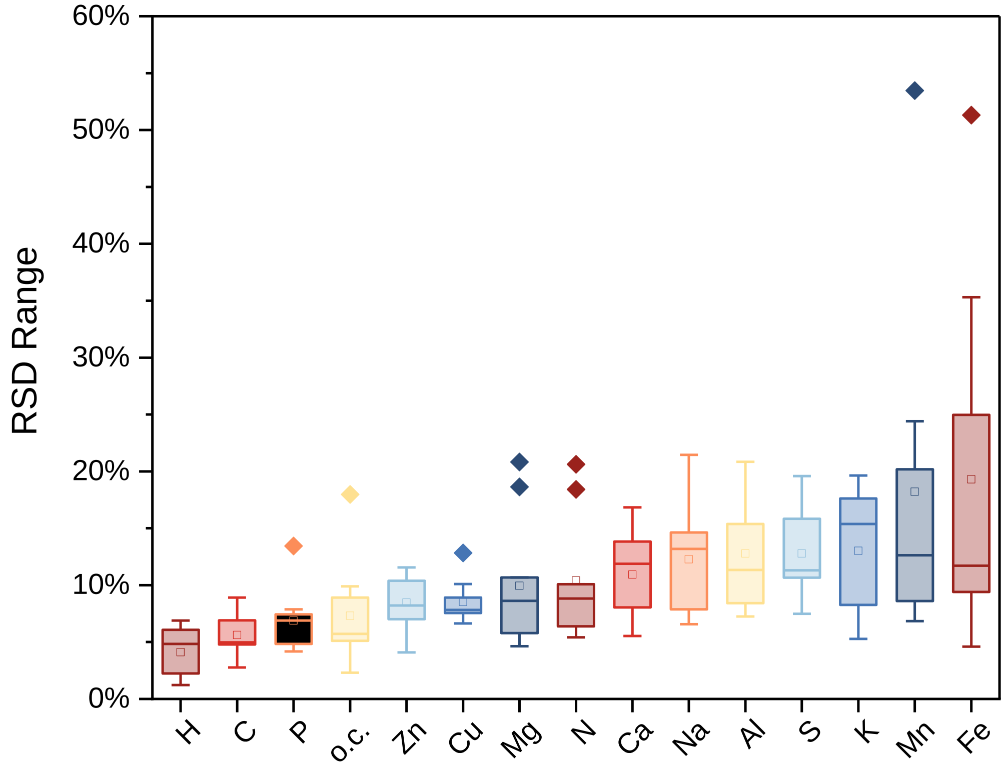


**Figure S3: Ranges of relative standard deviation (RSD) of matrix elements and organic content for the 9 WWTPs over the whole year**

RSDs were formed by analysis of monthly sewage sludge samples from 9 WWTPs a to i.

All annual RSDs (also trace elements) are shown in table S2, S4, S6, S8, S10, S12, S14, S16 and S18 (last column)

***Additional file 1*** *- Variation of the element composition of municipal sewage sludges in the context of new regulations on phosphorus recovery in Germany*

**Table S1: Certified reference material sewage sludges used for analysis and calibration limits**

| | | **CRM 029-50G** | | **CRM 031-40G** | | **Calibration** |
|---|---|---|---|---|---|---|
| | | **Certified** | **Range of recovery** | **Certified** | **Range of recovery** | **Determination limit** |
| As | [mg/kg] | 256 ± 9 | 100-111% | 290 ± 6 | 100% | 0.7 |
| Mo | [mg/kg] | 155 ± 4 | 96-100% | 112 ± 6 | 98-100% | 0.4 |
| Pb | [mg/kg] | 119 ± 5 | 80-86% | 129 ± 10 | 88-114% | 1.0 |
| Cu | [mg/kg] | 736 ± 33 | 100% | 487 ± 10 | 100-105% | 5.0 |
| Ni | [mg/kg] | 127 ± 4 | 73-78% | 94 ± 15 | 101-118% | 0.6 |
| K | [g/kg] | 4.9 ± 1.0 | 72-79% | 5.6 ± 0.5 | 70-97% | < 0.1 |
| Na | [g/kg] | 3.7 ± 0.3 | 94-100% | | | < 0.1 |
| Mg | [g/kg] | 11.9 ± 0.3 | 71-77% | 15.4 ± 0.9 | 72-85% | < 0.1 |
| Ca | [g/kg] | 38.0 ± 2.8 | 100% | 50.9 ± 1.9 | 89-98% | 0.4 |
| Cr | [mg/kg] | 353 ± 12 | 100% | 151 ± 8 | 96-105% | 0.8 |
| Mn | [mg/kg] | 264 ± 10 | 128% | 989 ± 123 | 98-104% | 3.2 |
| Fe | [g/kg] | 20.2 ± 1.3 | 100% | 32.7 ± 0.7 | 92-100% | 0.3 |
| Zn | [mg/kg] | 1.080 ± 20 | 96-100% | 967 ± 21 | 100-105% | 7.5 |
| Sn | [mg/kg] | 139 ± 9 | 117% | 156 ± 21 | 100% | 0.5 |
| Al | [g/kg] | | | 19.3 ± 1.5 | 93-97% | 0.2 |
| P | [g/kg] | 21.1 ± 2.6 | 100% | 21.5 ± 1.5 | 92-100% | 0.2 |

***Additional file 1*** *- Variation of the element composition of municipal sewage sludges in the context of new regulations on phosphorus recovery in Germany*

**Table S2: Total data for WWTP a, all analyzed values per sample**

| a | Sample | 1 | 2 | 3 | 4 | 5 | 6 | 7 | 8 | 9 | 10 | 11 | RSD |
|---|---|---|---|---|---|---|---|---|---|---|---|---|---|
| **Date** | | *Jul 19* | *Jul 19* | *Aug 19* | *Sep 19* | *Oct 19* | *Nov 19* | *Dec 19* | *Jan 20* | *Feb 20* | *May 20* | *Jun 20* | |
| **o.c.** | [%] | 81 | 82 | 76 | 77 | 80 | 80 | 82 | 79 | 81 | 80 | 79 | 2% |
| **C** | [g/kg] | 440.6 | 412.6 | 416.9 | 410.7 | 432.2 | 43.05 | 440.2 | 417.1 | 442.2 | 437.3 | 427.5 | 3% |
| **Ca** | [g/kg] | 22.2 | 24.5 | 23.6 | 23.0 | 25.2 | 24.0 | 24.3 | 25.9 | 24.4 | 25.7 | 21.2 | 6% |
| **P** | [g/kg] | 17.6 | 22.4 | 20.2 | 22.7 | 20.6 | 19.7 | 21.5 | 23.1 | 21.8 | 21.7 | 20.9 | 7% |
| $\frac{P_{month}}{P_{year}}$ | [%] | 84 | 106 | 96 | 107 | 97 | 93 | 102 | 110 | 103 | 103 | 99 | |
| **N** | [g/kg] | 39.9 | 41.5 | 39.3 | 43.0 | 41.6 | 40.3 | 44.5 | 42.1 | 46.1 | 44.0 | 45.5 | 5% |
| **H** | [g/kg] | 65.2 | 62.9 | 62.9 | 62.8 | 64.8 | 64.0 | 65.9 | 63.0 | 65.5 | 64.3 | 61.9 | 2% |
| **Mg** | [g/kg] | 2.5 | 2.2 | 2.0 | 2.2 | 2.3 | 2.2 | 2.3 | 2.3 | 2.3 | 2.3 | 2.1 | 6% |
| **K** | [g/kg] | 1.9 | 1.6 | 1.4 | 1.6 | 1.6 | 1.5 | 1.7 | 1.6 | 1.5 | 1.5 | 1.6 | 8% |
| **S** | [g/kg] | 8.7 | 14.6 | 20.7 | 20.8 | 16.9 | 15.6 | 15.8 | 17.9 | 15.4 | 17.0 | 13.8 | 21% |
| **Fe** | [g/kg] | 25.9 | 43.7 | 44.0 | 48.1 | 37.1 | 32.0 | 34.8 | 43.4 | 36.7 | 39.6 | 36.5 | 16% |
| **Al** | [g/kg] | 3.2 | 3.8 | 3.9 | 3.3 | 3.3 | 3.3 | 3.7 | 3.4 | 3.8 | 3.9 | 3.4 | 7% |
| **Mn** | [mg/kg] | 166 | 271 | 298 | 283 | 274 | 202 | 232 | 260 | 181 | 328 | 282 | 20% |
| **Na** | [mg/kg] | 872 | 697 | 681 | 694 | 738 | 753 | 752 | 708 | 709 | 736 | 600 | 9% |
| **Zn** | [mg/kg] | 742 | 588 | 599 | 630 | 537 | 569 | 642 | 517 | 611 | 506 | 538 | 12% |
| **Cu** | [mg/kg] | 430 | 521 | | | | 457 | 521 | 498 | 518 | 497 | 502 | 7% |
| **Pb** | [mg/kg] | 59 | 22 | 24 | 22 | 18 | 16 | 21 | 15 | 13 | 12 | 18 | 59% |
| **Cr** | [mg/kg] | 34 | 20 | 21 | 17 | 30 | 23 | 17 | 15 | 19 | 15 | 12 | 33% |
| **Ni** | [mg/kg] | 34 | 16 | 17 | 14 | 11 | 14 | 15 | 11 | 13 | 11 | 8 | 45% |
| **Sn** | [mg/kg] | 13 | 14 | 14 | 12 | 13 | 12 | 13 | 12 | 14 | 11 | | 9% |
| **Mo** | [mg/kg] | 3 | 7 | 7 | 6 | 4 | 3 | 4 | 6 | 4 | 5 | 6 | 28% |
| **As** | [mg/kg] | 7 | 6 | 7 | 6 | 4 | 4 | 5 | 5 | 4 | 8 | | 26% |

Organic content (o.c.; as loss of ignition at 550 °C) and relative standard deviation (monthly deviation divided by annual average) for all elements.

RSDs in the range of the RSD of phosphorus (6-8% RSD) are marked in green, higher RSDs in yellow and lower RSDs (higher constancy over the year) in red

***Additional file 1*** *- Variation of the element composition of municipal sewage sludges in the context of new regulations on phosphorus recovery in Germany*

**Table S3: Relative standard deviation data of the replicates for analyzed values of WWTP a**

| **WWTP a** | **RSD** | **1** | **2** | **3** | **4** | **5** | **6** | **7** | **8** | **9** | **10** | **11** |
|---|---|---|---|---|---|---|---|---|---|---|---|---|
| **Date** | | *Jul 19* | *Jul 19* | *Aug 19* | *Sep 19* | *Oct 19* | *Nov 19* | *Dec 19* | *Jan 20* | *Feb 20* | *May 20* | *Jun 20* |
| **o.c.** | [%] | < 0.5 | < 0.5 | 2 | < 0.5 | 1 | < 0.5 | < 0.5 | < 0.5 | < 0.5 | < 0.5 | < 0.5 |
| **Moisture** | [%] | 11 | 2 | 1 | 3 | < 0.5 | 2 | 3 | 1 | 1 | 3 | 5 |
| **C** | [%] | < 0.5 | < 0.5 | < 0.5 | < 0.5 | < 0.5 | < 0.5 | < 0.5 | < 0.5 | < 0.5 | < 0.5 | < 0.5 |
| **Ca** | [%] | 1 | 1 | 1 | 2 | 2 | 1 | 1 | 8 | < 0.5 | 2 | 3 |
| **P** | [%] | 1 | 1 | < 0.5 | 2 | 2 | 1 | 2 | 5 | < 0.5 | 1 | 2 |
| **N** | [%] | < 0.5 | < 0.5 | < 0.5 | < 0.5 | < 0.5 | < 0.5 | < 0.5 | < 0.5 | < 0.5 | < 0.5 | < 0.5 |
| **H** | [%] | 1 | 1 | < 0.5 | < 0.5 | < 0.5 | < 0.5 | < 0.5 | < 0.5 | < 0.5 | < 0.5 | < 0.5 |
| **Mg** | [%] | 1 | 3 | 1 | 2 | 3 | 1 | 1 | 5 | 1 | 1 | 3 |
| **K** | [%] | 1 | 3 | 1 | 2 | 2 | < 0.5 | 1 | 2 | < 0.5 | 3 | 2 |
| **S** | [%] | 3 | 2 | < 0.5 | 2 | 2 | 1 | 1 | 16 | < 0.5 | 2 | 1 |
| **Fe** | [%] | 11 | 3 | 1 | 1 | 3 | 1 | < 0.5 | 10 | < 0.5 | 1 | 3 |
| **Al** | [%] | 2 | 9 | 1 | 2 | 9 | 3 | 1 | 13 | 1 | 2 | 5 |
| **Mn** | [%] | 5 | 6 | < 0.5 | 2 | 1 | 2 | 1 | 18 | < 0.5 | 1 | 3 |
| **Na** | [%] | 6 | 7 | < 0.5 | < 0.5 | 1 | 1 | 2 | 7 | 4 | 5 | 2 |
| **Zn** | [%] | 6 | < 0.5 | 1 | 2 | 3 | 1 | 1 | 12 | 1 | 1 | 3 |
| **Cu** | [%] | 3 | 4 | | | | | 1 | 6 | < 0.5 | 1 | 2 |
| **Pb** | [%] | 2 | 4 | 3 | 1 | 4 | 1 | < 0.5 | 9 | 2 | 3 | 8 |
| **Cr** | [%] | 7 | 8 | 1 | 6 | 79 | 25 | 4 | 16 | 16 | 22 | 5 |
| **Ni** | [%] | 11 | 11 | 2 | 1 | 6 | 19 | 1 | 13 | 3 | < 0.5 | 5 |
| **Sn** | [%] | 2 | 9 | 5 | 3 | 9 | 1 | < 0.5 | 13 | 1 | 2 | |
| **Mo** | [%] | 4 | 2 | 1 | 3 | 2 | 6 | 1 | 22 | 3 | 3 | 4 |
| **As** | [%] | 3 | 6 | 4 | 2 | 8 | 3 | 1 | 7 | 4 | 3 | |

**Table S4: Total data for WWTP b, all analyzed values per sample**

| b | Sample | 1 | 2 | 3 | 4 | 5 | 6 | 7 | 8 | 9 | 10 | RSD |
|---|---|---|---|---|---|---|---|---|---|---|---|---|
| **Date** | | *Aug 19* | *Oct 19* | *Nov 19* | *Dec 19* | *Jan 20* | *Feb 20* | *Mar 20* | *May 20* | *Jun 20* | *Jul 20* | |
| **o.c.** | [%] | 41 | 40 | 39 | 37 | 39 | 41 | 37 | 42 | 44 | 38 | 6% |
| **C** | [g/kg] | 226.1 | 202.9 | 208.8 | 208.3 | 215.3 | 225.6 | 214.3 | 232.8 | 235.0 | 215.9 | 5% |
| **Ca** | [g/kg] | 132.1 | 162.6 | 151.6 | 162.7 | 159.2 | 149.4 | 170.9 | 155.5 | 135.9 | 141.0 | 8% |
| **P** | [g/kg] | 25.4 | 22.4 | 23.7 | 23.0 | 23.7 | 24.6 | 23.9 | 24.9 | 22.7 | 22.4 | 5% |
| $\frac{P_{month}}{P_{year}}$ | [%] | 107 | 95 | 100 | 97 | 100 | 104 | 101 | 105 | 96 | 95 | |
| **N** | [g/kg] | 27.8 | 24.8 | 26.4 | 26.1 | 28.1 | 29.6 | 28.0 | 30.5 | 29.3 | 26.6 | 6% |
| **H** | [g/kg] | 36.2 | 38.5 | 37.3 | 34.8 | 37.2 | 27.2 | 35.2 | 37.0 | 36.7 | 32.3 | 5% |
| **Mg** | [g/kg] | 8.7 | 8.1 | 7.9 | 9.2 | 9.4 | 9.3 | 9.3 | 9.8 | 8.9 | 9.0 | 6% |
| **K** | [g/kg] | 3.5 | 3.3 | 3.5 | 3.1 | 3.1 | 3.2 | 3.4 | 3.4 | | 3.1 | 5% |
| **S** | [g/kg] | 10.4 | 9.2 | 9.8 | 9.5 | 10.0 | 10.5 | 9.5 | 11.2 | 7.9 | 7.2 | 13% |
| **Fe** | [g/kg] | 45.3 | 45.0 | 47.9 | 47.0 | 46.5 | 46.3 | 44.2 | 38.0 | 35.4 | 44.4 | 9% |
| **Al** | [g/kg] | 18.9 | 15.5 | 15.7 | 14.1 | 14.2 | 13.4 | 13.5 | 13.2 | 11.6 | 11.0 | 16% |
| **Mn** | [mg/kg] | 348 | 305 | 321 | 304 | 325 | 347 | 349 | 266 | 248 | 254 | 13% |
| **Na** | [mg/kg] | 818 | 819 | 852 | 806 | 835 | 870 | 832 | 905 | 997 | 867 | 7% |
| **Zn** | [mg/kg] | 947 | 870 | 848 | 770 | 784 | 785 | 711 | 767 | 706 | 683 | 10% |
| **Cu** | [mg/kg] | 177 | 150 | 152 | 138 | 147 | 152 | 155 | 161 | 146 | 133 | 8% |
| **Pb** | [mg/kg] | 32 | 28 | 29 | 25 | 23 | 24 | 24 | 27 | 25 | 25 | 10% |
| **Cr** | [mg/kg] | 31 | 29 | 26 | 27 | 31 | 28 | 29 | 27 | 27 | 29 | 6% |
| **Ni** | [mg/kg] | 32 | 29 | 30 | 28 | 28 | 29 | 27 | 22 | 20 | 23 | 15% |
| **Sn** | [mg/kg] | 24 | 20 | 19 | 18 | 19 | 20 | 21 | 23 | | | 11% |
| **Mo** | [mg/kg] | 5 | 3 | 4 | 4 | 4 | 4 | 4 | 5 | 5 | 5 | 11% |
| **As** | [mg/kg] | 5 | 4 | 4 | 4 | 4 | 4 | 4 | 4 | | | 12% |

Organic content (o.c.; as loss of ignition at 550 °C) and relative standard deviation (monthly deviation divided by annual average) for all elements

RSDs in the range of the RSD of phosphorus (4-6% RSD) are marked in green, higher RSDs in yellow and lower RSDs (higher constancy over the year) in red

***Additional file 1*** *- Variation of the element composition of municipal sewage sludges in the context of new regulations on phosphorus recovery in Germany*

**Table S5: Relative standard deviation data of the replicates for analyzed values of WWTP b**

| **WWTP b** | **RSD** | **1** | **2** | **3** | **4** | **5** | **6** | **7** | **8** | **9** | **10** |
|---|---|---|---|---|---|---|---|---|---|---|---|
| **Date** | | *Aug 19* | *Oct 19* | *Nov 19* | *Dec 19* | *Jan 20* | *Feb 20* | *Mar 20* | *May 20* | *Jun 20* | *Jul 20* |
| **o.c.** | [%] | < 0.5 | < 0.5 | < 0.5 | 1 | < 0.5 | < 0.5 | < 0.5 | < 0.5 | < 0.5 | < 0.5 |
| **Moisture** | [%] | 2 | 3 | 2 | 4 | 3 | 1 | 4 | 3 | 1 | 1 |
| **C** | [%] | < 0.5 | < 0.5 | < 0.5 | < 0.5 | < 0.5 | < 0.5 | < 0.5 | < 0.5 | < 0.5 | < 0.5 |
| **Ca** | [%] | 2 | 1 | < 0.5 | < 0.5 | 1 | 2 | < 0.5 | 1 | 1 | 1 |
| **P** | [%] | 1 | 2 | 1 | 1 | < 0.5 | < 0.5 | 1 | 1 | 1 | < 0.5 |
| **N** | [%] | < 0.5 | < 0.5 | < 0.5 | < 0.5 | 1 | < 0.5 | 1 | < 0.5 | < 0.5 | < 0.5 |
| **H** | [%] | 2 | < 0.5 | < 0.5 | < 0.5 | < 0.5 | < 0.5 | < 0.5 | < 0.5 | < 0.5 | < 0.5 |
| **Mg** | [%] | 3 | < 0.5 | < 0.5 | < 0.5 | 1 | 2 | 1 | 1 | < 0.5 | < 0.5 |
| **K** | [%] | 11 | 4 | 1 | 1 | < 0.5 | 3 | < 0.5 | 8 | < 0.5 | 1 |
| **S** | [%] | 1 | 2 | 2 | 1 | 1 | 1 | 1 | 2 | < 0.5 | < 0.5 |
| **Fe** | [%] | 3 | 2 | 1 | 1 | 3 | 2 | 1 | 1 | 3 | < 0.5 |
| **Al** | [%] | 8 | 1 | 1 | 1 | 1 | 3 | 1 | 6 | 4 | < 0.5 |
| **Mn** | [%] | 4 | 3 | < 0.5 | 2 | 2 | 4 | < 0.5 | < 0.5 | < 0.5 | < 0.5 |
| **Na** | [%] | 4 | 2 | 1 | 1 | 2 | 5 | 1 | 1 | 1 | 4 |
| **Zn** | [%] | 1 | 2 | 1 | < 0.5 | 1 | 1 | 5 | 5 | 5 | 2 |
| **Cu** | [%] | 4 | 3 | 2 | 1 | 1 | 4 | 3 | 1 | < 0.5 | < 0.5 |
| **Pb** | [%] | 3 | 3 | 1 | 3 | 3 | 1 | 4 | 3 | 5 | 5 |
| **Cr** | [%] | 8 | 8 | 1 | 1 | 1 | 6 | < 0.5 | 5 | 1 | 4 |
| **Ni** | [%] | 7 | 4 | 4 | 7 | 9 | 9 | 2 | 2 | < 0.5 | 3 |
| **Sn** | [%] | < 0.5 | 1 | 1 | < 0.5 | 3 | 3 | 5 | 1 | | |
| **Mo** | [%] | 5 | 2 | 4 | 1 | 5 | 4 | 1 | 3 | < 0.5 | 1 |
| **As** | [%] | 5 | 1 | 2 | 14 | 2 | 3 | 12 | 6 | | |

***Additional file 1*** *- Variation of the element composition of municipal sewage sludges in the context of new regulations on phosphorus recovery in Germany*

**Table S6: Total data for WWTP c, all analyzed values per sample**

| c | | 1 | 2 | 3 | 4 | 5 | 6 | 7 | 8 | 9 | 10 | 11 | 12 | 13 | RSD |
|---|---|---|---|---|---|---|---|---|---|---|---|---|---|---|---|
| **Date** | | *Aug 19* | *Sep 19* | *Sep 19* | *Oct 19* | *Nov 19* | *Dec 19* | *Jan 20* | *Feb 20* | *Mar 20* | *Apr 20* | *May 20* | *Jun 20* | *Jul 20* | |
| **o.c.** | [%] | 33 | 28 | 30 | 34 | 31 | 29 | 28 | 31 | 32 | 33 | 33 | 30 | 24 | 9% |
| **C** | [g/kg] | 193.3 | 179.3 | 184.1 | 196.3 | 183.0 | 177.1 | 176.1 | 188.5 | 195.0 | 183.9 | 187.7 | 170.4 | 169.1 | 5% |
| **Ca** | [g/kg] | 199.9 | 222.2 | 211.3 | 188.3 | 200.2 | 211.0 | 217.9 | 211.1 | 182.3 | 202.4 | 201.5 | 215.7 | 206.1 | 6% |
| **P** | [g/kg] | 22.6 | 21.4 | 21.6 | 22.0 | 21.9 | 20.9 | 20.8 | 20.7 | 21.0 | 19.6 | 18.5 | 19.5 | 17.6 | 7% |
| $\frac{P_{month}}{P_{year}}$ | [%] | 110 | 104 | 105 | 106 | 106 | 101 | 101 | 100 | 102 | 95 | 90 | 94 | 86 | |
| **N** | [g/kg] | 16.8 | 14.6 | 14.3 | 17.1 | 15.6 | 16.0 | 16.3 | 17.6 | 24.1 | 22.2 | 22.9 | 22.2 | 19.8 | 18% |
| **H** | [g/kg] | | | | | | | | | 32.4 | 32.4 | 33.0 | 32.6 | 32.7 | 1% |
| **Mg** | [g/kg] | 7.6 | 7.7 | 9.3 | 8.2 | 8.8 | 8.8 | 8.3 | 7.5 | 7.8 | 8.4 | 8.6 | 9.9 | 9.1 | 9% |
| **K** | [g/kg] | 1.8 | 1.8 | 1.9 | 1.7 | 1.9 | 1.9 | 2.0 | 2.0 | 2.0 | 2.0 | 2.4 | 1.9 | 1.8 | 9% |
| **S** | [g/kg] | 7.6 | 7.1 | 7.1 | 7.8 | 7.3 | 7.2 | 7.1 | 7.4 | 7.3 | 7.1 | 7.0 | 6.9 | 4.7 | 11% |
| **Fe** | [g/kg] | 53.9 | 48.9 | 49.3 | 59.8 | 56.3 | 53.6 | 50.3 | 47.2 | 49.6 | 50.0 | 46.7 | 42.8 | 39.6 | 11% |
| **Al** | [g/kg] | 5.3 | 5.0 | 5.0 | 5.4 | 6.0 | 6.0 | 6.0 | 5.8 | 5.8 | 5.6 | 5.8 | 5.4 | 3.9 | 11% |
| **Mn** | [mg/kg] | 328 | 306 | 299 | 306 | 354 | 321 | 346 | 308 | 315 | 355 | 320 | 377 | 318 | 7% |
| **Na** | [mg/kg] | 664 | 643 | 679 | 620 | 680 | 650 | 655 | 606 | 568 | 597 | 762 | 625 | 586 | 8% |
| **Zn** | [mg/kg] | 545 | 496 | 495 | 548 | 528 | 498 | 506 | 512 | 545 | 476 | 486 | 491 | 409 | 7% |
| **Cu** | [mg/kg] | 244 | 225 | 223 | 249 | 235 | 222 | 220 | 220 | 229 | 205 | 214 | 212 | 181 | 8% |
| **Pb** | [mg/kg] | 28 | 25 | 26 | 27 | 26 | 25 | 24 | 22 | 23 | 21 | 20 | 19 | 27 | 11% |
| **Cr** | [mg/kg] | 25 | 26 | 20 | 28 | 25 | 22 | 20 | 18 | 20 | 24 | 22 | 18 | 15 | 17% |
| **Ni** | [mg/kg] | 16 | 14 | 14 | 16 | 15 | 14 | 14 | 13 | 15 | 19 | 16 | 13 | 10 | 14% |
| **Sn** | [mg/kg] | 17 | 16 | 16 | 18 | 16 | 15 | 14 | 14 | 15 | 12 | 13 | 13 | | 12% |
| **Mo** | [mg/kg] | 2 | 2 | 2 | 2 | 2 | 2 | 2 | 2 | 2 | 2 | 2 | 2 | 1 | 18% |
| **As** | [mg/kg] | 1 | <1 | 1 | <1 | <1 | <1 | <1 | 1 | 1 | 1 | 1 | 1 | | 47% |

Organic content (o.c.; as loss of ignition at 550 °C) and relative standard deviation (monthly deviation divided by annual average) for all elements

RSDs in the range of the RSD of phosphorus (6-8% RSD) are marked in green, higher RSDs in yellow and lower RSDs (higher constancy over the year) in red

***Additional file 1*** *- Variation of the element composition of municipal sewage sludges in the context of new regulations on phosphorus recovery in Germany*

**Table S7: Relative standard deviation data of the replicates for analyzed values of WWTP c**

| WWTP c | RSD | 1 | 2 | 3 | 4 | 5 | 6 | 7 | 8 | 9 | 10 | 11 | 12 | 13 |
|---|---|---|---|---|---|---|---|---|---|---|---|---|---|---|
| **Date** | | *Aug 19* | *Sep 19* | *Sep 19* | *Oct 19* | *Nov 19* | *Dec 19* | *Jan 20* | *Feb 20* | *Mar 20* | *Apr 20* | *May 20* | *Jun 20* | *Jul 20* |
| **o.c.** | [%] | < 0.5 | < 0.5 | 1 | < 0.5 | < 0.5 | < 0.5 | < 0.5 | < 0.5 | < 0.5 | < 0.5 | 1 | < 0.5 | < 0.5 |
| **Moisture** | [%] | 1 | 1 | 2 | < 0.5 | 5 | 3 | 2 | 3 | 2 | 4 | 4 | 2 | 3 |
| **C** | [%] | < 0.5 | < 0.5 | < 0.5 | < 0.5 | < 0.5 | < 0.5 | < 0.5 | < 0.5 | < 0.5 | < 0.5 | < 0.5 | < 0.5 | < 0.5 |
| **Ca** | [%] | 1 | 1 | < 0.5 | < 0.5 | < 0.5 | < 0.5 | 1 | < 0.5 | 1 | 1 | 1 | 1 | 1 |
| **P** | [%] | 1 | 1 | 1 | 6 | 1 | 2 | < 0.5 | < 0.5 | 1 | 1 | 3 | 2 | < 0.5 |
| **N** | [%] | 1 | < 0.5 | 1 | 1 | 1 | 1 | 1 | 2 | < 0.5 | < 0.5 | 1 | < 0.5 | 1 |
| **H** | [%] | | | | | | | | | 2 | < 0.5 | < 0.5 | < 0.5 | < 0.5 |
| **Mg** | [%] | 2 | 1 | 1 | 1 | 1 | 1 | 1 | < 0.5 | < 0.5 | 1 | 1 | 1 | < 0.5 |
| **K** | [%] | 2 | 2 | 1 | < 0.5 | 1 | < 0.5 | 3 | 1 | 1 | 1 | < 0.5 | 2 | 3 |
| **S** | [%] | 1 | < 0.5 | 1 | 2 | < 0.5 | 1 | 1 | < 0.5 | 1 | 1 | < 0.5 | 1 | 1 |
| **Fe** | [%] | 1 | < 0.5 | < 0.5 | 1 | 1 | 1 | 1 | < 0.5 | < 0.5 | 1 | < 0.5 | 2 | 1 |
| **Al** | [%] | 2 | 1 | < 0.5 | 2 | 1 | 1 | 3 | 1 | 1 | 1 | 3 | 1 | 2 |
| **Mn** | [%] | 2 | 1 | < 0.5 | 1 | 1 | 1 | 1 | < 0.5 | < 0.5 | 1 | < 0.5 | 2 | < 0.5 |
| **Na** | [%] | 2 | 1 | 2 | < 0.5 | < 0.5 | 3 | 2 | 2 | 2 | 1 | 1 | 2 | 2 |
| **Zn** | [%] | 1 | < 0.5 | < 0.5 | 1 | < 0.5 | 2 | 1 | 1 | 1 | 1 | < 0.5 | 5 | 2 |
| **Cu** | [%] | 2 | 1 | < 0.5 | < 0.5 | 1 | 1 | 1 | < 0.5 | 1 | 1 | 1 | 1 | < 0.5 |
| **Pb** | [%] | 1 | 1 | 1 | 1 | < 0.5 | 2 | 2 | < 0.5 | < 0.5 | 1 | 1 | 1 | 4 |
| **Cr** | [%] | 10 | 10 | 1 | 27 | 14 | 4 | 4 | 1 | 1 | 2 | 5 | 1 | 3 |
| **Ni** | [%] | 3 | 1 | 2 | 2 | 3 | 2 | 9 | < 0.5 | < 0.5 | < 0.5 | 2 | 4 | 1 |
| **Sn** | [%] | 2 | 2 | 1 | 2 | 1 | 5 | 3 | < 0.5 | 1 | 2 | 1 | 3 | |
| **Mo** | [%] | 3 | 2 | 6 | 2 | 2 | 1 | 3 | 1 | < 0.5 | 1 | 3 | 5 | 13 |
| **As** | [%] | 2 | 28 | 25 | 13 | 15 | 56 | 42 | 3 | 30 | 21 | 12 | 7 | |

***Additional file 1*** *- Variation of the element composition of municipal sewage sludges in the context of new regulations on phosphorus recovery in Germany*

**Table S8: Total data for WWTP d, all analyzed values per sample**

| d | Sample | 1 | 2 | 3 | 4 | 5 | 6 | 7 | 8 | 9 | 10 | RSD |
|---|---|---|---|---|---|---|---|---|---|---|---|---|
| **Date** | | *Aug 19* | *Oct 19* | *Nov 19* | *Dec 19* | *Jan 20* | *Feb 20* | *Mar 20* | *May 20* | *Jun 20* | *Jul 20* | |
| **o.c.** | [%] | 25 | 24 | 25 | 27 | 22 | 28 | 25 | 30 | 30 | 27 | 10% |
| **C** | [g/kg] | 158.4 | 169.0 | 169.1 | 176.4 | 152.8 | 171.7 | 173.7 | 178.9 | 177.1 | 174.1 | 5% |
| **Ca** | [g/kg] | 251.4 | 257.8 | 251.9 | 242.3 | 273.9 | 251.8 | 220.2 | 219.2 | 183.7 | 202.9 | 12% |
| **P** | [g/kg] | 16.8 | 17.6 | 18.1 | 18.3 | 16.1 | 18.3 | 17.6 | 18.3 | 17.1 | 17.1 | 4% |
| $\frac{P_{month}}{P_{year}}$ | [%] | 96 | 100 | 103 | 104 | 92 | 104 | 100 | 104 | 98 | 98 | |
| **N** | [g/kg] | 5.0 | 8.5 | 8.8 | 9.6 | 8.6 | 9.6 | 10.9 | 11.5 | 9.3 | 11.7 | 21% |
| **H** | [g/kg] | 31.7 | 32.2 | 32.4 | 32.8 | 31.9 | 33.6 | 33.0 | 33.0 | 33.5 | 31.5 | 2% |
| **Mg** | [g/kg] | 6.9 | 6.8 | 7.0 | 7.1 | 6.8 | 7.3 | 6.9 | 6.9 | 6.4 | 6.2 | 5% |
| **K** | [g/kg] | 1.2 | 1.3 | 1.3 | 1.4 | 1.6 | 1.8 | 2.2 | 1.8 | 1.6 | 1.5 | 20% |
| **S** | [g/kg] | 21.2 | 19.9 | 21.2 | 21.0 | 20.6 | 21.6 | 14.6 | 16.5 | 15.6 | 15.3 | 15% |
| **Fe** | [g/kg] | 37.4 | 34.2 | 37.0 | 37.6 | 35.7 | 36.8 | 36.0 | 39.2 | 36.7 | 33.4 | 5% |
| **Al** | [g/kg] | 4.7 | 4.9 | 4.9 | 4.9 | 4.5 | 5.0 | 4.5 | 4.3 | 3.8 | 3.6 | 11% |
| **Mn** | [mg/kg] | 244 | 254 | 260 | 247 | 239 | 254 | 262 | 246 | 214 | 216 | 7% |
| **Na** | [mg/kg] | 825 | 989 | 943 | 935 | 880 | 890 | 959 | 1.049 | 1.013 | 863 | 8% |
| **Zn** | [mg/kg] | 447 | 454 | 479 | 463 | 383 | 430 | 431 | 414 | 393 | 422 | 7% |
| **Cu** | [mg/kg] | 196 | 204 | 209 | 206 | 166 | 198 | 182 | 192 | 172 | 185 | 8% |
| **Pb** | [mg/kg] | 12 | 11 | 11 | 11 | 9 | 9 | 12 | 11 | 12 | 15 | 15% |
| **Cr** | [mg/kg] | 11 | 11 | 11 | 11 | 9 | 10 | 10 | 11 | 10 | 11 | 6% |
| **Ni** | [mg/kg] | 15 | 13 | 15 | 16 | 12 | 13 | 14 | 12 | 13 | 12 | 10% |
| **Sn** | [mg/kg] | 8 | 9 | 9 | 8 | 7 | 8 | 7 | 8 | | | 9% |
| **Mo** | [mg/kg] | 3 | 4 | 4 | 4 | 2 | 3 | 3 | 3 | 3 | 2 | 19% |
| **As** | [mg/kg] | <1 | <1 | <1 | <1 | 1 | <1 | <1 | <1 | | | 35% |

Organic content (o.c.; as loss of ignition at 550 °C) and relative standard deviation (monthly deviation divided by annual average) for all elements

RSDs in the range of the RSD of phosphorus (3-5% RSD) are marked in green, higher RSDs in yellow and lower RSDs (higher constancy over the year) in red

***Additional file 1*** *- Variation of the element composition of municipal sewage sludges in the context of new regulations on phosphorus recovery in Germany*

**Table S9: Relative standard deviation data of the replicates for analyzed values of WWTP d**

| WWTP d | RSD | 1 | 2 | 3 | 4 | 5 | 6 | 7 | 8 | 9 | 10 |
|---|---|---|---|---|---|---|---|---|---|---|---|
| **Date** | | *Aug 19* | *Oct 19* | *Nov 19* | *Dec 19* | *Jan 20* | *Feb 20* | *Mar 20* | *May 20* | *Jun 20* | *Jul 20* |
| **o.c.** | [%] | < 0.5 | 1 | < 0.5 | < 0.5 | < 0.5 | < 0.5 | 1 | < 0.5 | < 0.5 | < 0.5 |
| **Moisture** | [%] | 3 | 2 | 1 | 2 | 3 | 2 | 5 | 5 | 2 | < 0.5 |
| **C** | [%] | < 0.5 | 1 | < 0.5 | < 0.5 | < 0.5 | < 0.5 | < 0.5 | < 0.5 | < 0.5 | < 0.5 |
| **Ca** | [%] | 1 | < 0.5 | 2 | < 0.5 | < 0.5 | < 0.5 | 3 | 1 | 2 | 1 |
| **P** | [%] | 2 | 1 | 2 | < 0.5 | < 0.5 | < 0.5 | 2 | < 0.5 | 2 | 1 |
| **N** | [%] | 4 | 3 | 1 | < 0.5 | < 0.5 | 1 | 1 | < 0.5 | < 0.5 | < 0.5 |
| **H** | [%] | 3 | 3 | < 0.5 | < 0.5 | < 0.5 | < 0.5 | < 0.5 | < 0.5 | < 0.5 | 1 |
| **Mg** | [%] | 2 | 1 | 2 | < 0.5 | < 0.5 | < 0.5 | 3 | 1 | 1 | < 0.5 |
| **K** | [%] | 2 | 1 | 3 | < 0.5 | 1 | < 0.5 | < 0.5 | 2 | < 0.5 | 1 |
| **S** | [%] | 2 | 1 | 2 | 1 | < 0.5 | < 0.5 | 2 | < 0.5 | < 0.5 | 1 |
| **Fe** | [%] | 2 | < 0.5 | 2 | < 0.5 | < 0.5 | < 0.5 | 2 | 1 | 5 | 9 |
| **Al** | [%] | 3 | 1 | 2 | < 0.5 | < 0.5 | 1 | 5 | 4 | < 0.5 | 1 |
| **Mn** | [%] | 1 | < 0.5 | 2 | 1 | 1 | < 0.5 | 3 | 2 | 1 | 1 |
| **Na** | [%] | 3 | < 0.5 | 1 | 3 | 3 | 3 | 1 | 4 | 2 | 5 |
| **Zn** | [%] | 3 | < 0.5 | 2 | 1 | < 0.5 | 1 | 2 | 1 | 2 | 1 |
| **Cu** | [%] | 1 | < 0.5 | 2 | < 0.5 | < 0.5 | 1 | 2 | 3 | 1 | 1 |
| **Pb** | [%] | 6 | 4 | 4 | 2 | 4 | < 0.5 | 4 | 3 | < 0.5 | 1 |
| **Cr** | [%] | 5 | < 0.5 | 2 | 2 | 6 | 12 | 5 | 4 | 2 | < 0.5 |
| **Ni** | [%] | 4 | 1 | 3 | 5 | 1 | 3 | 7 | 5 | 2 | 4 |
| **Sn** | [%] | 8 | 1 | < 0.5 | 3 | 1 | 5 | 2 | 3 | | |
| **Mo** | [%] | 3 | < 0.5 | 1 | < 0.5 | 2 | 8 | 4 | 1 | 7 | 2 |
| **As** | [%] | 86 | 17 | 4 | 82 | 7 | 214 | 3 | 29 | | |

***Additional file 1*** *- Variation of the element composition of municipal sewage sludges in the context of new regulations on phosphorus recovery in Germany*

**Table S10: Total data for WWTP e. all analyzed values per sample**

| e | | 1 | 2 | 3 | 4 | 5 | 6 | 7 | 8 | 9 | 10 | 11 | 12 | RSD |
|---|---|---|---|---|---|---|---|---|---|---|---|---|---|---|
| Date | | Oct 19 | Nov 19 | Dec 19 | Jan 20 | Feb 20 | Mar 20 | Apr 20 | May 20 | Jun 20 | Jul 20 | Aug 20 | Sep 20 | |
| o.c. | [%] | 64 | 64 | 64 | 68 | 67 | 60 | 66 | 65 | 66 | 65 | 61 | 65 | 4% |
| C | [g/kg] | 331.6 | 432.2 | 349.4 | 364.2 | 348.8 | 307.4 | 348.8 | 343.8 | 339.4 | 337.5 | 311.0 | 332.3 | 5% |
| Ca | [g/kg] | 13.0 | 12.2 | 11.5 | 11.2 | 11.7 | 11.2 | 13.1 | 12.1 | 12.3 | 12.6 | 14.1 | 14.1 | 8% |
| P | [g/kg] | 23.4 | 25.4 | 24.8 | 22.9 | 24.0 | 23.9 | 25.8 | 24.7 | 24.9 | 25.1 | 27.1 | 26.3 | 5% |
| $\frac{P_{month}}{P_{year}}$ | [%] | 94 | 102 | 100 | 92 | 97 | 96 | 104 | 99 | 100 | 101 | 109 | 106 | |
| N | [g/kg] | 37.5 | 41.7 | 42.4 | 46.2 | 47.4 | 42.4 | 45.1 | 46.3 | 53.4 | 45.5 | 42.4 | 42.8 | 9% |
| H | [g/kg] | 51.4 | 52.9 | 54.1 | 56.1 | 53.8 | 49.3 | 52.9 | 53.3 | 46.8 | 51.8 | 49.2 | 52.6 | 5% |
| Mg | [g/kg] | 3.7 | 3.4 | 3.1 | 3.4 | 3.7 | 4.6 | 3.5 | 3.5 | 3.6 | 3.9 | 4.2 | 3.9 | 11% |
| K | [g/kg] | 4.3 | 4.4 | 4.2 | 4.5 | 4.7 | 6.7 | 4.2 | | | | | 4.4 | 18% |
| S | [g/kg] | 5.4 | 5.5 | 5.4 | 5.1 | 5.4 | 4.9 | 6.4 | 5.4 | 5.1 | 5.3 | 6.2 | 5.8 | 8% |
| Fe | [g/kg] | 28.0 | 26.2 | 25.3 | 23.0 | 24.1 | 27.9 | 23.9 | 22.0 | 20.8 | 21.3 | 21.5 | 19.6 | 12% |
| Al | [g/kg] | 23.6 | 25.0 | 25.2 | 22.8 | 24.8 | 33.0 | 26.7 | 26.1 | 25.3 | 27.1 | 30.9 | 31.4 | 12% |
| Mn | [mg/kg] | 366 | 351 | 328 | 213 | 218 | 282 | 211 | 204 | 195 | 202 | 224 | 265 | 24% |
| Na | [mg/kg] | 990 | 949 | 954 | 944 | 917 | 819 | 1.165 | 934 | 780 | 1.022 | 1.060 | 1.238 | 13% |
| Zn | [mg/kg] | 1.088 | 1.089 | 1.011 | 948 | 1.012 | 1.136 | 890 | 991 | 975 | 1.026 | 1.115 | 1.035 | 7% |
| Cu | [mg/kg] | 164 | 163 | 157 | 176 | 183 | 182 | 146 | 155 | 151 | 151 | 162 | 152 | 8% |
| Pb | [mg/kg] | 57 | 55 | 52 | 48 | 52 | 77 | 50 | 44 | 43 | 44 | 46 | 48 | 18% |
| Cr | [mg/kg] | 42 | 40 | 37 | 35 | 36 | 52 | 35 | 34 | 29 | 31 | 33 | 32 | 17% |
| Ni | [mg/kg] | 21 | 20 | 19 | 18 | 19 | 28 | 21 | 20 | 18 | 19 | 21 | 19 | 12% |
| Sn | [mg/kg] | 21 | 21 | 20 | 26 | 27 | 25 | 23 | | | | | | 11% |
| Mo | [mg/kg] | 4 | 4 | 4 | 4 | 4 | 4 | 4 | 5 | 5 | 5 | 7 | 5 | 19% |
| As | [mg/kg] | 4 | 3 | 4 | 3 | 3 | 8 | 4 | | | | | | 38% |

Organic content (o.c.; as loss of ignition at 550 °C) and relative standard deviation (monthly deviation divided by annual average) for all elements

RSDs in the range of the RSD of phosphorus (4-6% RSD) are marked in green. higher RSDs in yellow and lower RSDs (higher constancy over the year) in red

***Additional file 1*** *- Variation of the element composition of municipal sewage sludges in the context of new regulations on phosphorus recovery in Germany*

**Table S11: Relative standard deviation data of the replicates for analyzed values of WWTP e**

| **WWTP e** | **RSD** | **1** | **2** | **3** | **4** | **5** | **6** | **7** | **8** | **9** | **10** | **11** | **12** |
|---|---|---|---|---|---|---|---|---|---|---|---|---|---|
| **Date** | | *Oct 19* | *Nov 19* | *Dec 19* | *Jan 20* | *Feb 20* | *Mar 20* | *Apr 20* | *May 20* | *Jun 20* | *Jul 20* | *Aug 20* | *Sep 20* |
| **o.c.** | [%] | < 0.5 | < 0.5 | < 0.5 | < 0.5 | < 0.5 | < 0.5 | < 0.5 | < 0.5 | < 0.5 | < 0.5 | 1 | < 0.5 |
| **Moisture** | [%] | 2 | 1 | 1 | 2 | 1 | 1 | 1 | 2 | 4 | 1 | 1 | 2 |
| **C** | [%] | < 0.5 | < 0.5 | < 0.5 | < 0.5 | < 0.5 | < 0.5 | < 0.5 | < 0.5 | < 0.5 | < 0.5 | < 0.5 | < 0.5 |
| **Ca** | [%] | 1 | < 0.5 | 2 | 1 | < 0.5 | < 0.5 | 1 | 3 | < 0.5 | 1 | < 0.5 | < 0.5 |
| **P** | [%] | 3 | 1 | 2 | < 0.5 | 3 | 4 | 1 | 4 | 1 | 1 | 1 | 1 |
| **N** | [%] | 1 | < 0.5 | 1 | < 0.5 | < 0.5 | 1 | < 0.5 | < 0.5 | 4 | 1 | < 0.5 | < 0.5 |
| **H** | [%] | 4 | 1 | < 0.5 | < 0.5 | 1 | 2 | 3 | 3 | 4 | 1 | 1 | < 0.5 |
| **Mg** | [%] | 1 | 1 | 2 | < 0.5 | < 0.5 | 2 | < 0.5 | 1 | 2 | 1 | < 0.5 | < 0.5 |
| **K** | [%] | 1 | < 0.5 | 1 | 2 | 5 | 5 | 1 | | | | | 1 |
| **S** | [%] | 1 | 1 | 2 | < 0.5 | < 0.5 | 4 | 1 | 2 | 1 | < 0.5 | 1 | < 0.5 |
| **Fe** | [%] | 1 | 2 | 1 | < 0.5 | < 0.5 | 4 | < 0.5 | 3 | 4 | 4 | 1 | 1 |
| **Al** | [%] | 1 | 1 | 1 | 1 | < 0.5 | 3 | 1 | 1 | 3 | 1 | < 0.5 | 1 |
| **Mn** | [%] | 2 | 2 | 1 | < 0.5 | < 0.5 | 3 | 1 | 1 | 2 | < 0.5 | < 0.5 | 2 |
| **Na** | [%] | 2 | 1 | 3 | 1 | < 0.5 | 4 | < 0.5 | 2 | 3 | 3 | 3 | 1 |
| **Zn** | [%] | 1 | 5 | 1 | < 0.5 | 1 | 5 | < 0.5 | 1 | 1 | < 0.5 | < 0.5 | 1 |
| **Cu** | [%] | 2 | 2 | 1 | < 0.5 | < 0.5 | 3 | < 0.5 | 2 | < 0.5 | < 0.5 | 2 | 1 |
| **Pb** | [%] | < 0.5 | 2 | 1 | < 0.5 | < 0.5 | 3 | < 0.5 | 2 | 5 | 2 | 2 | < 0.5 |
| **Cr** | [%] | 1 | 3 | 2 | 2 | < 0.5 | 6 | 3 | 6 | 8 | 4 | < 0.5 | 3 |
| **Ni** | [%] | 1 | 2 | 1 | 1 | < 0.5 | 3 | 4 | 2 | 1 | 2 | < 0.5 | 3 |
| **Sn** | [%] | 1 | 1 | 1 | 2 | < 0.5 | 4 | < 0.5 | | | | | |
| **Mo** | [%] | 2 | 9 | 4 | 1 | 2 | 4 | 1 | 1 | 11 | 3 | 4 | 2 |
| **As** | [%] | 1 | 12 | 6 | 8 | 15 | 2 | 3 | | | | | |

***Additional file 1*** *- Variation of the element composition of municipal sewage sludges in the context of new regulations on phosphorus recovery in Germany*

**Table S12: Total data for WWTP f. all analyzed values per sample**

| f | | 1 | 2 | 3 | 4 | 5 | 6 | 7 | 8 | 9 | 10 | 11 | 12 | RSD |
|---|---|---|---|---|---|---|---|---|---|---|---|---|---|---|
| Date | | *Oct 19* | *Nov 19* | *Dec 19* | *Jan 20* | *Feb 20* | *Mar 20* | *Apr 20* | *May 20* | *Jun 20* | *Jul 20* | *Aug 20* | *Sep 20* | |
| o.c. | [%] | 63 | 64 | 63 | 65 | 65 | 65 | 64 | 63 | 60 | 57 | 57 | 57 | 5% |
| C | [g/kg] | 318.2 | 329.0 | 315.0 | 336.6 | 339.7 | | 329.1 | 317.8 | 309.9 | 291.6 | 294.1 | 289.1 | 6% |
| Ca | [g/kg] | 19.4 | 18.9 | 17.5 | 15.9 | 15.6 | 14.8 | 17.7 | 20.3 | 21.4 | 21.8 | 23.0 | 20.0 | 14% |
| P | [g/kg] | 26.0 | 25.4 | 23.2 | 21.8 | 21.4 | 22.0 | 25.8 | 24.8 | 24.4 | 23.6 | 25.6 | 23.0 | 7% |
| $\frac{P_{month}}{P_{year}}$ | [%] | 109 | 106 | 97 | 91 | 89 | 92 | 108 | 104 | 102 | 99 | 107 | 96 | |
| N | [g/kg] | 49.3 | 50.5 | 48.9 | 51.7 | 50.2 | | 49.9 | 48.3 | 45.8 | 43.6 | 44.3 | 44.3 | 6% |
| H | [g/kg] | 54.0 | 54.8 | 55.4 | 55.5 | 55.4 | | 55.2 | 53.9 | 50.9 | 48.5 | 48.6 | 47.3 | 6% |
| Mg | [g/kg] | 4.7 | 4.7 | 3.9 | 4.1 | 4.2 | 4.2 | 4.6 | 4.9 | 6.1 | 6.6 | 6.6 | 6.5 | 21% |
| K | [g/kg] | 4.0 | 4.2 | 3.9 | 4.8 | 4.6 | 4.8 | 5.8 | 5.7 | 5.7 | 6.2 | 5.9 | 5.6 | 16% |
| S | [g/kg] | 5.2 | 5.1 | 4.7 | 4.5 | 4.4 | 4.3 | 5.1 | 5.1 | 5.6 | 5.3 | 5.5 | 4.9 | 8% |
| Fe | [g/kg] | 8.9 | 9.0 | 8.1 | 9.0 | 9.6 | 10.0 | 9.3 | 9.2 | 14.2 | 12.1 | 11.4 | 17.1 | 25% |
| Al | [g/kg] | 61.8 | 61.5 | 66.1 | 53.3 | 52.4 | 56.1 | 64.5 | 66.9 | 59.0 | 60.5 | 60.1 | 48.7 | 10% |
| Mn | [mg/kg] | 299 | 277 | 218 | 208 | 216 | 212 | 209 | 232 | 276 | 325 | 319 | 311 | 18% |
| Na | [mg/kg] | 1.123 | 1.155 | 1.121 | 1.016 | 953 | 918 | 1.217 | 1.458 | 1.619 | 1.623 | 1.695 | 1.351 | 21% |
| Zn | [mg/kg] | 1.073 | 1.088 | 1.052 | 1.009 | 1.096 | 1.037 | 1.126 | 1.109 | 1.099 | 1.056 | 1.010 | 993 | 4% |
| Cu | [mg/kg] | 229 | 229 | 251 | 203 | 198 | 205 | 247 | 262 | 241 | 240 | 222 | 216 | 9% |
| Pb | [mg/kg] | 65 | 63 | 63 | 59 | 62 | 65 | 30 | 21 | 44 | 48 | 42 | 62 | 29% |
| Cr | [mg/kg] | 35 | 35 | 30 | 31 | 32 | 34 | 36 | 39 | 48 | 45 | 44 | 41 | 15% |
| Ni | [mg/kg] | 29 | 29 | 22 | 24 | 27 | 26 | 28 | 30 | 38 | 35 | 36 | 36 | 17% |
| Mo | [mg/kg] | 5 | 5 | 3 | 3 | 3 | 3 | 4 | 4 | 6 | 6 | 7 | 8 | 37% |

Organic content (o.c.; as loss of ignition at 550 °C) and relative standard deviation (monthly deviation divided by annual average) for all elements

RSDs in the range of the RSD of phosphorus (6-8% RSD) are marked in green. higher RSDs in yellow and lower RSDs (higher constancy over the year) in red

*Additional file 1* - *Variation of the element composition of municipal sewage sludges in the context of new regulations on phosphorus recovery in Germany*

**Table S13: Relative standard deviation data of the replicates for analyzed values of WWTP f**

| WWTP f | RSD | 1 | 2 | 3 | 4 | 5 | 6 | 7 | 8 | 9 | 10 | 11 | 12 |
|---|---|---|---|---|---|---|---|---|---|---|---|---|---|
| **Date** | | *Oct 19* | *Nov 19* | *Dec 19* | *Jan 20* | *Feb 20* | *Mar 20* | *Apr 20* | *May 20* | *Jun 20* | *Jul 20* | *Aug 20* | *Sep 20* |
| **o.c.** | [%] | < 0.5 | < 0.5 | < 0.5 | 1 | < 0.5 | < 0.5 | < 0.5 | < 0.5 | < 0.5 | < 0.5 | 1 | 1 |
| **Moisture** | [%] | 3 | 3 | 2 | 5 | 4 | | 2 | 2 | 3 | 5 | 5 | 7 |
| **C** | [%] | < 0.5 | 1 | < 0.5 | 1 | < 0.5 | | 1 | < 0.5 | < 0.5 | < 0.5 | < 0.5 | < 0.5 |
| **Ca** | [%] | 1 | 1 | < 0.5 | 3 | 3 | | 2 | 2 | 2 | 2 | 4 | 1 |
| **P** | [%] | 1 | 3 | 1 | 2 | 2 | 3 | 2 | < 0.5 | 2 | 1 | 3 | < 0.5 |
| **N** | [%] | 2 | 2 | 1 | 2 | 1 | | 1 | 1 | 1 | 1 | 1 | < 0.5 |
| **H** | [%] | 1 | 1 | 1 | < 0.5 | < 0.5 | | 1 | 1 | < 0.5 | 1 | < 0.5 | 1 |
| **Mg** | [%] | 3 | 2 | 1 | 3 | 1 | 1 | 2 | 2 | 2 | 2 | 3 | 2 |
| **K** | [%] | 6 | 3 | 2 | 3 | 8 | 2 | 1 | 3 | < 0.5 | 2 | 4 | 2 |
| **S** | [%] | < 0.5 | 1 | < 0.5 | 2 | < 0.5 | < 0.5 | 3 | < 0.5 | 1 | 1 | 3 | 4 |
| **Fe** | [%] | 2 | 1 | 6 | 1 | 1 | < 0.5 | 5 | < 0.5 | 31 | 1 | 3 | 48 |
| **Al** | [%] | 1 | 1 | 1 | 2 | 1 | < 0.5 | 2 | < 0.5 | 2 | 1 | 3 | 3 |
| **Mn** | [%] | 2 | 1 | 1 | 1 | 3 | 1 | 1 | < 0.5 | 1 | 2 | 3 | 2 |
| **Na** | [%] | 1 | 1 | 1 | 3 | 1 | < 0.5 | 3 | 3 | 2 | 2 | 2 | 2 |
| **Zn** | [%] | 2 | 1 | 2 | 1 | 2 | 6 | 7 | 1 | 1 | 1 | 3 | 13 |
| **Cu** | [%] | < 0.5 | < 0.5 | 3 | 2 | 3 | < 0.5 | < 0.5 | 1 | 4 | 2 | 2 | 19 |
| **Pb** | [%] | 3 | 2 | 5 | 3 | 2 | < 0.5 | 1 | 2 | 8 | 4 | 3 | 40 |
| **Cr** | [%] | 5 | 2 | < 0.5 | 3 | 2 | < 0.5 | 2 | < 0.5 | 3 | 2 | 4 | 5 |
| **Ni** | [%] | < 0.5 | < 0.5 | 2 | 2 | 3 | 1 | 2 | 1 | 1 | 2 | 3 | 4 |
| **Mo** | [%] | 3 | 2 | 12 | 2 | 3 | 2 | 2 | 2 | 7 | 2 | 2 | 46 |

**Table S14: Total data for WWTP g. all analyzed values per sample**

| g | Sample | 1 | 2 | 3 | 4 | 5 | 6 | 7 | 8 | 9 | RSD |
|---|---|---|---|---|---|---|---|---|---|---|---|
| **Date** | | *Oct 19* | *Feb 20* | *Mar 20* | *Apr 20* | *May 20* | *Jun 20* | *Jul 20* | *Aug 20* | *Sep 20* | |
| **o.c.** | [%] | 65 | 78 | 70 | 76 | 74 | 74 | 72 | 70 | 77 | 6% |
| **C** | [g/kg] | 329.4 | 422.8 | 397.0 | 417.3 | 416.0 | 400.5 | 391.3 | 400.5 | 402.2 | 7% |
| **Ca** | [g/kg] | 2.3 | 7.9 | 7.2 | 10.2 | 9.6 | 9.7 | 9.7 | 10.9 | 11.8 | 17% |
| **P** | [g/kg] | 24.2 | 17.4 | 16.2 | 20.3 | 19.3 | 18.8 | 20.0 | 20.8 | 24.2 | 13% |
| $\frac{P_{month}}{P_{year}}$ | [%] | 120 | 87 | 81 | 101 | 96 | 93 | 100 | 103 | 120 | |
| **N** | [g/kg] | 53.5 | 75.0 | 68.9 | 77.7 | 75.3 | 72.5 | 71.3 | 72.0 | 70.8 | 10% |
| **H** | [g/kg] | 51.8 | 65.2 | 59.5 | 65.3 | 64.5 | 61.3 | 59.2 | 60.6 | 62.8 | 7% |
| **Mg** | [g/kg] | 3.1 | 3.4 | 3.4 | 3.6 | 3.5 | 3.5 | 3.6 | 3.4 | 4.2 | 9% |
| **K** | [g/kg] | 3.8 | 6.2 | 4.8 | 5.2 | 4.9 | 4.6 | 4.2 | 3.5 | 6.0 | 19% |
| **S** | [g/kg] | 5.9 | 5.3 | 4.6 | 5.8 | 5.8 | 5.7 | 7.4 | 9.0 | 6.3 | 21% |
| **Fe** | [g/kg] | 48.5 | 16.2 | 15.0 | 18.2 | 16.0 | 16.2 | 17.9 | 19.1 | 18.1 | 51% |
| **Al** | [g/kg] | 10.5 | 8.2 | 8.5 | 7.1 | 6.5 | 6.2 | 6.6 | 6.4 | 6.4 | 20% |
| **Mn** | [mg/kg] | 538 | 158 | 167 | 154 | 143 | 157 | 268 | 311 | 253 | 53% |
| **Na** | [mg/kg] | 656 | 562 | 508 | 820 | 617 | 564 | 567 | 670 | 615 | 15% |
| **Zn** | [mg/kg] | 872 | 815 | 755 | 677 | 678 | 693 | 688 | 673 | 752 | 10% |
| **Cu** | [mg/kg] | 192 | 166 | 139 | 144 | 150 | 145 | 185 | 191 | 164 | 13% |
| **Pb** | [mg/kg] | 51 | 50 | 45 | 35 | 33 | 33 | 46 | 43 | 45 | 16% |
| **Cr** | [mg/kg] | 38 | 26 | 27 | 25 | 23 | 24 | 30 | 29 | 23 | 17% |
| **Ni** | [mg/kg] | 23 | 19 | 18 | 19 | 18 | 17 | 22 | 21 | 23 | 11% |
| **Mo** | [mg/kg] | 3 | 5 | 2 | 2 | 3 | 2 | 4 | 5 | 3 | 38% |

Organic content (o.c.; as loss of ignition at 550 °C) and relative standard deviation (monthly deviation divided by annual average) for all elements

RSDs in the range of the RSD of phosphorus (12-14% RSD) are marked in green. higher RSDs in yellow and lower RSDs (higher constancy over the year) in red

***Additional file 1*** *- Variation of the element composition of municipal sewage sludges in the context of new regulations on phosphorus recovery in Germany*

**Table S15: Relative standard deviation data of the replicates for analyzed values of WWTP g**

| **WWTP g** | **RSD** | **1** | **2** | **3** | **4** | **5** | **6** | **7** | **8** | **9** |
|---|---|---|---|---|---|---|---|---|---|---|
| **Date** | | *Oct 19* | *Feb 20* | *Mar 20* | *Apr 20* | *May 20* | *Jun 20* | *Jul 20* | *Aug 20* | *Sep 20* |
| **o.c.** | [%] | < 0.5 | < 0.5 | < 0.5 | < 0.5 | 2 | 1 | < 0.5 | 1 | < 0.5 |
| **Moisture** | [%] | < 0.5 | 2 | 2 | 5 | 6 | 2 | < 0.5 | 3 | 1 |
| **C** | [%] | < 0.5 | 1 | < 0.5 | < 0.5 | < 0.5 | < 0.5 | < 0.5 | < 0.5 | < 0.5 |
| **Ca** | [%] | 2 | 1 | 1 | 1 | 2 | < 0.5 | 5 | 3 | 2 |
| **P** | [%] | < 0.5 | 4 | 1 | 3 | 1 | 2 | 4 | 2 | 2 |
| **N** | [%] | 1 | 1 | < 0.5 | 1 | < 0.5 | 1 | < 0.5 | < 0.5 | < 0.5 |
| **H** | [%] | 1 | 1 | 1 | < 0.5 | < 0.5 | < 0.5 | 1 | 1 | < 0.5 |
| **Mg** | [%] | < 0.5 | 2 | 1 | < 0.5 | 1 | 1 | 2 | 6 | 2 |
| **K** | [%] | 10 | 2 | < 0.5 | < 0.5 | 1 | < 0.5 | 3 | 9 | 3 |
| **S** | [%] | < 0.5 | 2 | < 0.5 | < 0.5 | 1 | 1 | 2 | 4 | 1 |
| **Fe** | [%] | 2 | 11 | < 0.5 | 1 | 1 | 2 | 1 | 4 | 5 |
| **Al** | [%] | 13 | 2 | 1 | 2 | 2 | 1 | 1 | 11 | 2 |
| **Mn** | [%] | 1 | 4 | < 0.5 | 1 | 2 | 1 | 3 | 3 | 9 |
| **Na** | [%] | 3 | 4 | 3 | 5 | 1 | 3 | 3 | 7 | 3 |
| **Zn** | [%] | 6 | 7 | < 0.5 | 2 | 3 | 6 | 2 | 3 | 3 |
| **Cu** | [%] | 2 | 10 | < 0.5 | 2 | 2 | 1 | 4 | 2 | 5 |
| **Pb** | [%] | 1 | 8 | 1 | 2 | 1 | 1 | < 0.5 | 4 | 2 |
| **Cr** | [%] | 10 | < 0.5 | < 0.5 | 1 | < 0.5 | 5 | 4 | 7 | 6 |
| **Ni** | [%] | < 0.5 | 2 | < 0.5 | 3 | < 0.5 | < 0.5 | 8 | 6 | 38 |
| **Mo** | [%] | 2 | 34 | 2 | 2 | < 0.5 | 5 | 1 | 2 | 7 |

***Additional file 1*** *- Variation of the element composition of municipal sewage sludges in the context of new regulations on phosphorus recovery in Germany*

**Table S16: Total data for WWTP h. all analyzed values per sample**

| h | | 1 | 2 | 3 | 4 | 5 | 6 | 7 | 8 | 9 | 10 | 11 | 12 | RSD |
|---|---|---|---|---|---|---|---|---|---|---|---|---|---|---|
| **Date** | | *Oct 19* | *Nov 19* | *Dec 19* | *Jan 20* | *Feb 20* | *Mar 20* | *Apr 20* | *May 20* | *Jun 20* | *Jul 20* | *Aug 20* | *Sep 20* | |
| **o.c.** | [%] | 25 | 21 | 24 | 23 | 18 | 23 | 23 | 26 | 31 | 30 | 32 | 32 | 18% |
| **C** | [g/kg] | 166.5 | 160.0 | 172.2 | 168.8 | 162.3 | 170.1 | 166.7 | 181.9 | 193.0 | 194.5 | 201.5 | 204.6 | 9% |
| **Ca** | [g/kg] | 193.5 | 216.3 | 200.1 | 204.0 | 219.4 | 211.4 | 216.0 | 203.9 | 178.9 | 147.3 | 148.9 | 161.2 | 14% |
| **P** | [g/kg] | 18.0 | 17.6 | 19.0 | 18.3 | 16.4 | 16.9 | 17.5 | 16.9 | 18.4 | 18.1 | 19.2 | 20.0 | 6% |
| $\frac{P_{month}}{P_{year}}$ | [%] | 100 | 98 | 105 | 102 | 91 | 94 | 97 | 94 | 102 | 100 | 106 | 111 | |
| **N** | [g/kg] | 17.2 | 17.4 | 18.6 | 18.8 | 17.5 | 18.5 | 18.2 | 18.9 | 20.3 | 20.5 | 21.4 | 21.6 | 8% |
| **H** | [g/kg] | 30.5 | 31.6 | 30.8 | 31.1 | 30.4 | 31.1 | 30.1 | 31.9 | 32.4 | 32.2 | 31.8 | 32.2 | 2% |
| **Mg** | [g/kg] | 6.0 | 5.7 | 5.7 | 5.6 | 5.6 | 6.1 | 5.9 | 6.2 | 6.5 | 5.9 | 6.3 | 6.5 | 5% |
| **K** | [g/kg] | 1.9 | 2.3 | 2.4 | 2.0 | 2.0 | | | | | 2.1 | 2.4 | | 8% |
| **S** | [g/kg] | 4.7 | 3.4 | 3.6 | 4.6 | 4.3 | 3.4 | 3.6 | 3.9 | 4.2 | 4.2 | 4.4 | 4.3 | 11% |
| **Fe** | [g/kg] | 41.7 | 44.8 | 47.8 | 44.6 | 40.7 | 42.3 | 41.5 | 40.8 | 43.5 | 34.8 | 34.4 | 39.1 | 9% |
| **Al** | [g/kg] | 8.3 | 8.4 | 8.1 | 7.5 | 7.8 | 10.0 | 9.1 | 9.2 | 10.1 | 9.1 | 9.7 | 10.8 | 11% |
| **Mn** | [mg/kg] | 300 | 261 | 248 | 246 | 265 | 284 | 263 | 288 | 314 | 284 | 302 | 316 | 9% |
| **Na** | [mg/kg] | 561 | 530 | 558 | 529 | 480 | 425 | 448 | 526 | 534 | 618 | 662 | 739 | 16% |
| **Zn** | [mg/kg] | 617 | 592 | 611 | 593 | 547 | 598 | 523 | 583 | 648 | 621 | 718 | 644 | 8% |
| **Cu** | [mg/kg] | 164 | 146 | 155 | 138 | 128 | 149 | 145 | 145 | 162 | 164 | 178 | 179 | 10% |
| **Pb** | [mg/kg] | 23 | 21 | 20 | 17 | 17 | 21 | 19 | 23 | 26 | 29 | 36 | 34 | 26% |
| **Cr** | [mg/kg] | 30 | 29 | 29 | 26 | 25 | 29 | 27 | 30 | 31 | 28 | 31 | 34 | 8% |
| **Ni** | [mg/kg] | 19 | 17 | 17 | 18 | 18 | 20 | 19 | 20 | 24 | 18 | 20 | 21 | 10% |
| **Sn** | [mg/kg] | 16 | 13 | 13 | 12 | 11 | 12 | 12 | 13 | 15 | | | | 11% |
| **Mo** | [mg/kg] | 1 | 1 | 1 | 1 | 1 | 1 | 1 | 1 | 2 | 2 | 2 | 2 | 32% |
| **As** | [mg/kg] | 3 | 1 | 1 | 2 | 2 | 3 | 2 | 2 | 2 | | | | 23% |

Organic content (o.c.; as loss of ignition at 550 °C) and relative standard deviation (monthly deviation divided by annual average) for all elements

RSDs in the range of the RSD of phosphorus (5-7% RSD) are marked in green. higher RSDs in yellow and lower RSDs (higher constancy over the year) in red

***Additional file 1*** *- Variation of the element composition of municipal sewage sludges in the context of new regulations on phosphorus recovery in Germany*

**Table S17: Relative standard deviation data of the replicates for analyzed values of WWTP h**

| **WWTP h** | **RSD** | **1** | **2** | **3** | **4** | **5** | **6** | **7** | **8** | **9** | **10** | **11** | **12** |
|---|---|---|---|---|---|---|---|---|---|---|---|---|---|
| **Date** | | *Oct 19* | *Nov 19* | *Dec 19* | *Jan 20* | *Feb 20* | *Mar 20* | *Apr 20* | *May 20* | *Jun 20* | *Jul 20* | *Aug 20* | *Sep 20* |
| **o.c.** | [%] | 2 | < 0.5 | < 0.5 | < 0.5 | < 0.5 | < 0.5 | < 0.5 | < 0.5 | < 0.5 | < 0.5 | < 0.5 | < 0.5 |
| **Moisture** | [%] | 3 | 2 | 4 | 5 | 3 | 6 | 6 | 5 | 2 | 1 | 4 | 3 |
| **C** | [%] | < 0.5 | < 0.5 | < 0.5 | < 0.5 | < 0.5 | < 0.5 | < 0.5 | < 0.5 | < 0.5 | < 0.5 | < 0.5 | < 0.5 |
| **Ca** | [%] | 1 | < 0.5 | 1 | 1 | < 0.5 | < 0.5 | 1 | 4 | < 0.5 | < 0.5 | 2 | 4 |
| **P** | [%] | 1 | 3 | 2 | 3 | < 0.5 | 1 | 1 | 2 | < 0.5 | < 0.5 | 1 | 1 |
| **N** | [%] | < 0.5 | 1 | 1 | 1 | 1 | 1 | 2 | 1 | 1 | < 0.5 | 1 | 1 |
| **H** | [%] | 3 | 1 | < 0.5 | < 0.5 | < 0.5 | < 0.5 | 3 | < 0.5 | < 0.5 | < 0.5 | < 0.5 | < 0.5 |
| **Mg** | [%] | 1 | 1 | 1 | 1 | < 0.5 | 1 | 1 | 3 | 1 | < 0.5 | 1 | 4 |
| **K** | [%] | 4 | 7 | 1 | 7 | 1 | < 0.5 | < 0.5 | < 0.5 | < 0.5 | 1 | < 0.5 | < 0.5 |
| **S** | [%] | 2 | 1 | 3 | 4 | 1 | 2 | 1 | 4 | 1 | 1 | 1 | 1 |
| **Fe** | [%] | < 0.5 | 1 | 1 | 3 | < 0.5 | 1 | 1 | 3 | 1 | < 0.5 | 1 | 1 |
| **Al** | [%] | 5 | 3 | 3 | 4 | 1 | 4 | 6 | 4 | 8 | 1 | 1 | 10 |
| **Mn** | [%] | 1 | 1 | 1 | 2 | < 0.5 | 1 | 1 | 2 | 1 | < 0.5 | < 0.5 | 1 |
| **Na** | [%] | 3 | 7 | 2 | 4 | 4 | 3 | 3 | 12 | 5 | 1 | 2 | 1 |
| **Zn** | [%] | < 0.5 | < 0.5 | 2 | 3 | < 0.5 | 1 | < 0.5 | 3 | 1 | 1 | 1 | 3 |
| **Cu** | [%] | 1 | 1 | 2 | 3 | < 0.5 | < 0.5 | 2 | 3 | 1 | < 0.5 | 1 | 2 |
| **Pb** | [%] | 3 | 3 | 3 | 1 | 1 | < 0.5 | 1 | 4 | 3 | < 0.5 | 2 | < 0.5 |
| **Cr** | [%] | 2 | 4 | 5 | 1 | 2 | 3 | 3 | 5 | 5 | 3 | < 0.5 | 4 |
| **Ni** | [%] | 2 | 7 | 7 | 1 | < 0.5 | 1 | 1 | 6 | 14 | 1 | 1 | 1 |
| **Sn** | [%] | < 0.5 | 2 | 1 | 3 | 1 | 8 | 2 | 3 | < 0.5 | | | |
| **Mo** | [%] | 2 | 4 | 9 | 2 | 1 | 4 | 10 | 1 | 1 | 2 | 2 | 1 |
| **As** | [%] | 6 | 20 | 24 | 12 | 17 | 19 | 5 | 11 | 16 | | | |

***Additional file 1*** *- Variation of the element composition of municipal sewage sludges in the context of new regulations on phosphorus recovery in Germany*

**Table S18: Total data for WWTP i. all analyzed values per sample**

| i | | 1 | 2 | 3 | 4 | 5 | 6 | 7 | 8 | 9 | 10 | 11 | 12 | RSD |
|---|---|---|---|---|---|---|---|---|---|---|---|---|---|---|
| **Date** | | *Nov 19* | *Dec 19* | *Jan 20* | *Feb 20* | *Mar 20* | *Apr 20* | *May 20* | *Jun 20* | *Jul 20* | *Aug 20* | *Sep 20* | *Oct 20* | |
| **o.c.** | [%] | 58 | 63 | 65 | 63 | 63 | 61 | 61 | 61 | 61 | 55 | 57 | 51 | 7% |
| **C** | [g/kg] | | 341.5 | 348.4 | 353.5 | 343.2 | 330.9 | 323.2 | 326.5 | 302.9 | 305.5 | 312.0 | 280.6 | 7% |
| **Ca** | [g/kg] | 36.4 | 32.5 | 26.4 | 27.0 | 24.8 | 25.5 | 26.5 | 31.7 | 35.1 | 32.7 | 33.9 | 25.9 | 14% |
| **P** | [g/kg] | 21.1 | 22.9 | 20.7 | 22.2 | 22.1 | 25.0 | 24.9 | 25.2 | 24.5 | 24.8 | 25.0 | 20.7 | 8% |
| $\frac{P_{month}}{P_{year}}$ | [%] | 91 | 98 | 89 | 95 | 95 | 108 | 107 | 108 | 106 | 107 | 107 | 89 | |
| **N** | [g/kg] | | 52.7 | 56.9 | 56.5 | 55.9 | 50.7 | 49.9 | 48.7 | 45.3 | 45.8 | 46.6 | 41.6 | 10% |
| **H** | [g/kg] | | 52.1 | 53.1 | 54.2 | 52.9 | 51.4 | 50.3 | 51.0 | 47.3 | 47.9 | 48.6 | 43.6 | 6% |
| **Mg** | [g/kg] | 6.6 | 6.4 | 7.4 | 8.2 | 8.0 | 5.2 | 4.6 | 5.0 | 5.8 | 5.8 | 6.0 | 5.5 | 19% |
| **K** | [g/kg] | 3.7 | 3.6 | 3.8 | 3.8 | 4.1 | 2.9 | | | | | 4.2 | 5.0 | 15% |
| **S** | [g/kg] | 9.6 | 10.1 | 10.1 | 9.7 | 9.5 | 9.3 | 9.6 | 9.3 | 9.6 | 8.8 | 9.5 | 11.7 | 7% |
| **Fe** | [g/kg] | 1.2 | 34.2 | 24.6 | 21.8 | 22.6 | 53.6 | 57.4 | 37.7 | 30.7 | 28.2 | 27.6 | 24.3 | 35% |
| **Al** | [g/kg] | 19.1 | 18.9 | 17.5 | 18.0 | 20.0 | 16.8 | 17.7 | 24.4 | 23.6 | 27.8 | 23.3 | 25.9 | 18% |
| **Mn** | [mg/kg] | 975 | 827 | 809 | 881 | 852 | 751 | 709 | 703 | 705 | 695 | 717 | 657 | 12% |
| **Na** | [mg/kg] | 657 | 469 | 536 | 553 | 497 | 460 | 473 | 554 | 712 | 679 | 624 | 622 | 14% |
| **Zn** | [mg/kg] | 848 | 826 | 806 | 818 | 793 | 703 | 700 | 750 | 761 | 783 | 768 | 1.035 | 11% |
| **Cu** | [mg/kg] | 368 | 365 | 341 | 351 | 339 | 325 | 316 | 329 | 314 | 320 | 334 | 398 | 7% |
| **Pb** | [mg/kg] | 46 | 42 | 44 | 44 | 46 | 42 | 44 | 37 | 33 | 32 | 38 | 46 | 12% |
| **Cr** | [mg/kg] | 56 | 51 | 48 | 49 | 51 | 47 | 48 | 48 | 50 | 51 | 46 | 54 | 6% |
| **Ni** | [mg/kg] | 43 | 42 | 46 | 45 | 44 | 37 | 32 | 31 | 31 | 33 | 30 | 64 | 24% |
| **Mo** | [mg/kg] | 18 | 18 | 19 | 19 | 17 | 16 | 16 | 16 | 13 | 13 | 13 | 16 | 14% |

Organic content (o.c.; as loss of ignition at 550 °C) and relative standard deviation (monthly deviation divided by annual average) for all elements

RSDs in the range of the RSD of phosphorus (7-9% RSD) are marked in green. higher RSDs in yellow and lower RSDs (higher constancy over the year) in red

*__Additional file 1__ - Variation of the element composition of municipal sewage sludges in the context of new regulations on phosphorus recovery in Germany*

**Table S19: Relative standard deviation data of the replicates for analyzed values of WWTP i**

| **WWTP i** | **RSD** | **1** | **2** | **3** | **4** | **5** | **6** | **7** | **8** | **9** | **10** | **11** | **12** |
|---|---|---|---|---|---|---|---|---|---|---|---|---|---|
| **Date** | | *Nov 19* | *Dec 19* | *Jan 20* | *Feb 20* | *Mar 20* | *Apr 20* | *May 20* | *Jun 20* | *Jul 20* | *Aug 20* | *Sep 20* | *Oct 20* |
| **o.c.** | [%] | < 0.5 | < 0.5 | 1 | < 0.5 | < 0.5 | < 0.5 | < 0.5 | < 0.5 | 1 | < 0.5 | < 0.5 | < 0.5 |
| **Moisture** | [%] | 6 | 3 | 4 | 2 | 5 | 4 | 2 | 2 | 6 | 2 | 6 | 5 |
| **C** | [%] | | < 0.5 | < 0.5 | < 0.5 | < 0.5 | < 0.5 | < 0.5 | < 0.5 | < 0.5 | < 0.5 | < 0.5 | < 0.5 |
| **Ca** | [%] | 3 | < 0.5 | 1 | 2 | 3 | 2 | 2 | 2 | < 0.5 | < 0.5 | 1 | 1 |
| **P** | [%] | 3 | 2 | 3 | < 0.5 | 2 | 2 | < 0.5 | 1 | 1 | 3 | 1 | < 0.5 |
| **N** | [%] | | < 0.5 | < 0.5 | < 0.5 | 1 | < 0.5 | 2 | 2 | 1 | < 0.5 | < 0.5 | 1 |
| **H** | [%] | | < 0.5 | < 0.5 | < 0.5 | < 0.5 | < 0.5 | 2 | < 0.5 | 1 | < 0.5 | < 0.5 | < 0.5 |
| **Mg** | [%] | 3 | < 0.5 | < 0.5 | 1 | 2 | 1 | 1 | < 0.5 | < 0.5 | 1 | 1 | < 0.5 |
| **K** | [%] | 7 | 9 | 10 | 4 | 3 | 1 | | | | | < 0.5 | < 0.5 |
| **S** | [%] | 2 | 1 | 2 | 1 | 2 | 2 | < 0.5 | 2 | 1 | < 0.5 | < 0.5 | 1 |
| **Fe** | [%] | 2 | 2 | 2 | 2 | 2 | 2 | < 0.5 | 2 | 1 | 8 | 2 | < 0.5 |
| **Al** | [%] | 2 | 5 | 6 | 1 | 3 | 2 | 1 | 2 | < 0.5 | < 0.5 | 1 | 1 |
| **Mn** | [%] | 3 | 2 | 1 | 4 | 3 | 2 | 2 | 4 | < 0.5 | 3 | 1 | 2 |
| **Na** | [%] | 2 | 4 | 1 | 2 | 2 | 1 | 2 | 6 | 1 | < 0.5 | 2 | 1 |
| **Zn** | [%] | 2 | 1 | 2 | 1 | 2 | 2 | 1 | < 0.5 | 1 | 4 | 1 | 1 |
| **Cu** | [%] | 3 | 2 | 2 | 3 | 2 | 2 | 2 | 3 | < 0.5 | 2 | < 0.5 | 1 |
| **Pb** | [%] | 2 | < 0.5 | < 0.5 | 2 | < 0.5 | 2 | 7 | 4 | 3 | 1 | < 0.5 | 1 |
| **Cr** | [%] | 1 | 1 | 1 | 3 | 4 | 3 | 1 | 5 | 3 | 4 | 2 | 1 |
| **Ni** | [%] | 3 | 1 | 2 | 5 | 3 | 2 | 2 | 4 | 1 | 3 | 2 | 1 |
| **Mo** | [%] | 2 | 1 | 3 | 3 | 2 | 2 | 1 | 4 | 1 | 2 | < 0.5 | 1 |

**Table S20: Share of in neutral ammonia citrate (NAC) extractable phosphorus ($P_{NAC}$) in several sewage sludge samples and molar ratios of P and potential precipitation agents Fe and Al**

| WWTP | Sample no. | Date | P | $P_{NAC}$ | Share of $P_{NAC}$ | Molar ratio Fe:P | Molar ratio Al:P | (Fe+Al):P | P elimination |
|---|---|---|---|---|---|---|---|---|---|
| | | | *[g/kg]* | *[g/kg]* | *[%]* | | | | |
| a | 4 | Sep 19 | 22.7 | 19.5 | 86 | 1.2 | 0.1 | 1.3 | EBPR. $FeCl_3$ |
| | 5 | Oct 19 | 20.6 | 15.7 | 76 | 1.0 | 0.2 | 1.2 | |
| | 8 | Jan 20 | 23.1 | 19.4 | 84 | 1.0 | 0.2 | 1.2 | |
| b | 2 | Oct 19 | 22.4 | 19.9 | 89 | 1.1 | 0.8 | 1.9 | $FeCl_3$. $AlCl_3$ |
| | 9 | Jun 20 | 22.7 | 20.7 | 91 | 0.9 | 0.6 | 1.5 | |
| c | 1 | Aug 19 | 22.6 | 20.3 | 90 | 1.2 | 0.3 | 1.5 | $FeCl_3$. $AlCl_3$ |
| | 13 | Jul 20 | 17.6 | 16.7 | 95 | 1.3 | 0.3 | 1.6 | |
| d | 3 | Nov 19 | 18.1 | 16.1 | 89 | 1.1 | 0.3 | 1.4 | EBPR. $FeCl_3$ |
| | 9 | Jun 20 | 17.1 | 17.1 | 100 | 1.2 | 0.2 | 1.4 | |
| e | 1 | Oct 19 | 23.4 | 20.6 | 88 | 0.7 | 1.2 | 1.8 | $FeCl_3$. sodium aluminate |
| f | 9 | Jun 20 | 24.4 | 18.8 | 77 | 0.3 | 2.8 | 3.1 | Sodium aluminate |
| | 11 | Aug 20 | 25.6 | 19.2 | 75 | 0.2 | 2.7 | 2.9 | |
| g | 2 | Feb 20 | 17.4 | 11.5 | 66 | 0.5 | 0.6 | 1.1 | EBPR. $FeCl_3$ |
| | 8 | Aug 20 | 24.2 | 17.9 | 74 | 0.5 | 0.4 | 0.9 | |
| h | 2 | Nov 19 | 17.6 | 16.7 | 95 | 1.4 | 0.5 | 2.0 | EBPR. $FeCl_3$ |
| i | 2 | Nov 19 | 22.9 | 19.7 | 86 | 0.8 | 1.0 | 1.9 | $FeCl_3$ |
| | 6 | Mar 20 | 22.1 | 17.9 | 81 | 0.6 | 1.0 | 1.6 | |
